# Machines vs. Ensembles: Effective MAPK Signaling through Heterogeneous Sets of Protein Complexes

**DOI:** 10.1371/journal.pcbi.1003278

**Published:** 2013-10-10

**Authors:** Ryan Suderman, Eric J. Deeds

**Affiliations:** 1 Center for Bioinformatics, University of Kansas, Lawrence, Kansas, United States of America; 2 Department of Molecular Biosciences, University of Kansas, Lawrence, Kansas, United States of America; Fox Chase Cancer Center, United States of America

## Abstract

Despite the importance of intracellular signaling networks, there is currently no consensus regarding the fundamental nature of the protein complexes such networks employ. One prominent view involves stable *signaling machines* with well-defined quaternary structures. The combinatorial complexity of signaling networks has led to an opposing perspective, namely that signaling proceeds via heterogeneous *pleiomorphic ensembles* of transient complexes. Since many hypotheses regarding network function rely on how we conceptualize signaling complexes, resolving this issue is a central problem in systems biology. Unfortunately, direct experimental characterization of these complexes has proven technologically difficult, while combinatorial complexity has prevented traditional modeling methods from approaching this question. Here we employ rule-based modeling, a technique that overcomes these limitations, to construct a model of the yeast pheromone signaling network. We found that this model exhibits significant ensemble character while generating reliable responses that match experimental observations. To contrast the ensemble behavior, we constructed a model that employs hierarchical assembly pathways to produce scaffold-based signaling machines. We found that this machine model could not replicate the experimentally observed *combinatorial inhibition* that arises when the scaffold is overexpressed. This finding provides evidence against the hierarchical assembly of machines in the pheromone signaling network and suggests that machines and ensembles may serve distinct purposes *in vivo*. In some cases, e.g. core enzymatic activities like protein synthesis and degradation, machines assembled via hierarchical energy landscapes may provide functional stability for the cell. In other cases, such as signaling, ensembles may represent a form of weak linkage, facilitating variation and plasticity in network evolution. The capacity of ensembles to signal effectively will ultimately shape how we conceptualize the function, evolution and engineering of signaling networks.

## Author Summary

Intracellular signaling networks are central to a cell's ability to adapt to its environment. Developing the capacity to effectively manipulate such networks would have a wide range of applications, from cancer therapy to synthetic biology. This requires a thorough understanding of the mechanisms of signal transduction, particularly the kinds of protein complexes that are formed during transmission of extracellular information to the nucleus. Traditionally, signaling complexes have been largely perceived (albeit often implicitly) as machine-like structures. However, the number of molecular complexes that could theoretically be formed by complex signaling networks is astronomically large. This has led to the *pleiomorphic ensemble* hypothesis, which posits that diverse and rapidly changing sets of transient protein complexes can transmit and process information. Our goal was to use computational approaches, specifically rule-based modeling, to test these hypotheses. We constructed a model of the prototypical yeast mating pathway and found significant ensemble-like behavior. Our results thus demonstrated that ensembles can in fact transmit extracellular signals with minimal noise. Additionally, a comparison of this model with one tailored to generate machine-like complexes displayed notable phenotypic differences, revealing potential advantages for ensemble-like signaling. Our demonstration that ensembles can function effectively will have a significant impact on how we conceptualize signaling and other processes inside cells.

## Introduction

**Figure 1:**
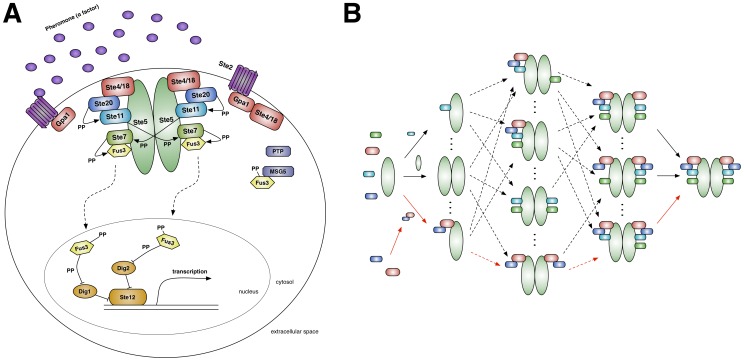
The yeast pheromone MAPK network. (A) A typical representation of the cascade. Pheromone (α-factor) stimulates G-protein activation via a GPCR (purple and red). The subsequent recruitment of the scaffold to the membrane enables the kinase phosphorylation cascade (blue and green), ultimately activating the MAPK, Fus3 (yellow), and regulating mating-related genes (orange). (B) Scaffold-based species potentially generated during our model's phosphorylation cascade (color coded to Fig. 1A). Solid arrows represent association events between either two monomers or a monomer and oligomer. Dashed arrows indicate a series of these association events. Red arrows indicate possible assembly pathways for the decamer (far right) in the machine model. Note that this is a very small sample of the entire set of scaffold-based signaling species and their possible interactions.

Much of our reasoning about the function of biological systems relies on the formation of multi-subunit protein complexes [1]. In some cases, such as the ribosome and the proteasome, these complexes take the form of intricate molecular machines with well-defined quaternary structures [2]–[4]. The overall structure of complexes formed during signal transduction, however, is considerably less clear. There are a few well-characterized signaling machines, like the apoptosome, and some have argued that the majority of structures produced by signaling networks would have a machine-like character [5], [6]. Most of the complexes formed during signal transmission and processing have not had their global three-dimensional structures experimentally determined, however, and as such we currently do not know the extent to which signaling occurs via machines [7]. Despite this uncertainty, the machine-like perspective on signaling complexes is pervasive in the literature, if often implicit; for instance, one commonly represents signaling networks graphically by drawing large complexes in which all of the relevant proteins interact simultaneously [8]–[14] (Fig. 1A). Although such diagrams are often presented as compact summaries of a set of interactions, they are certainly evocative of a machine-like structure, and lead naturally to analogies between signaling complexes and highly ordered objects such as circuit boards [7], [9].

One issue that complicates this machine-based picture is the fact that the protein interaction networks that underlie cellular signaling exhibit considerable *combinatorial complexity*; that is, they can (theoretically) generate anywhere from millions to 10^20^ or more unique molecular species [7], [15]–[17]. For example, even a single PDGF receptor dimer has ∼10^5^ possible phosphorylation states, many of which could be (stably) occupied by any given molecule [7], [18]. A similar problem arises in protein folding: a polypeptide chain could theoretically adopt so many conformations that it is *a priori* difficult to understand how a protein folds quickly and stably into a single native structure [16], [19], [20]. Proteins have evolved energy landscapes with specific features in order to overcome this problem (which is known as the “Levinthal paradox”). In order to assemble well-defined signaling machines, signaling networks would similarly need to evolve specific “chemical potential landscapes” in order to drive the system to a specific set of quaternary structures [16], [19].

Mayer *et al.* have speculated, however, that signaling networks might not need to assemble machine-like structures at all in order to function [7]. This “pleiomorphic ensemble” hypothesis posits that heterogeneous mixtures of complexes drive cellular responses to external signals. Early work, based on systems of Ordinary Differential Equations (ODEs) that considered a few hundred molecular species, indicated that more diffuse “network” models of signaling could generate reasonable signaling behavior [21], [22]. The dearth of computational methods that can handle combinatorially complex networks has made it difficult to fully test the ensemble hypothesis in realistic networks, however [16]. As such, it is currently unclear if ensembles could even produce reliable responses to signals, or if there is any functional or evolutionary difference between networks that employ ensembles vs. machines.

Over the past 10 years, a set of rule-based methods have been developed that allow one to model the behavior of biological systems without an *a priori* reduction in the set of possible species that can be formed [11], [16], [21], [23], [24]. Given a model consisting of a specific set of protein interaction rules, we can exactly sample sets of protein complexes (or “conformations”) from the astronomically large set of all possible complexes the model can generate. In this work we employed these methods to investigate the possibility of signaling via ensembles *in silico*. We focused on the pheromone response network (Fig. 1A), one of multiple mitogen-activated protein kinase (MAPK) cascades in *Saccharomyces cerevisiae*. This thoroughly characterized signaling cascade involves the scaffold protein Ste5, which is thought to be a nucleation point for the formation of signaling complexes (Fig. 1B) and prevent crosstalk [8]–[10]. Since similar MAPK cascades are found in eukaryotic cells from yeast to humans [25], this network represents an excellent model system for exploring the influence of combinatorial complexity on signaling dynamics.

In our initial model, we included only those interactions (and their requisite molecular contexts) that have been explicitly characterized experimentally. We found that this model is able to fit available data on the response of the network to pheromone, despite exhibiting significant ensemble character. We also constructed an alternative set of rules that could assemble a scaffold-based signaling machine, similar to those typically drawn to graphically summarize the cascade [8]–[14] (Fig. 1A). Although this model does fit some of the available data, we found that it could not replicate the “combinatorial inhibition” of the pathway observed at high levels of Ste5 overexpression [12], [26]; instead, it displayed considerable robustness to such changes. We also demonstrated that TAP/MS, a common technique for experimentally determining the components of “molecular machines” via binary interactions [27], [28], could not distinguish between the complexes formed in these two models, despite their radically different character. Direct experimental tests of the ensemble hypothesis thus require the application of assays that can measure three-way or higher-order interactions, such as fragment complementation, fluorescence triple correlation spectroscopy or single-molecule approaches [29]–[35]. Our findings indicate that ensembles can indeed reliably transmit and process extracellular information, and their inherent plasticity in response to perturbations like scaffold overexpression implies that they may play a role in facilitating the evolutionary variation of signaling systems within cells [36].

## Results

### Constructing a model of pheromone signaling in yeast

A summary of the molecular interactions underlying the yeast pheromone response network may be found in Fig. 1A. Briefly, the signaling cascade is initiated by the interaction between extracellular pheromone molecules and a G-protein coupled receptor (GPCR), which induces dissociation between the α subunit (Gpa1) and βγ subunits (the Ste4-Ste18 complex, hereafter referred to as Ste4) of the G-protein [37]. Ste4 then recruits the scaffold protein, Ste5, which dimerizes, binds numerous kinases (Ste20, Ste11, Ste7) and promotes a phosphorylation cascade resulting in dual-phosphorylation and activation of the MAPK, Fus3 [38], [39]. As mentioned above, the vast majority of graphical depictions of this cascade involve simultaneous binding of all requisite proteins to Ste5 (Fig. 1A) [8]–[14], however to our knowledge there is no explicit experimental evidence that such a large scaffold-based complex is actually formed during signaling. Active Fus3 then translocates to the nucleus, regulating the expression of numerous mating-related genes via the transcription factor Ste12 [10].

**Figure 2:**
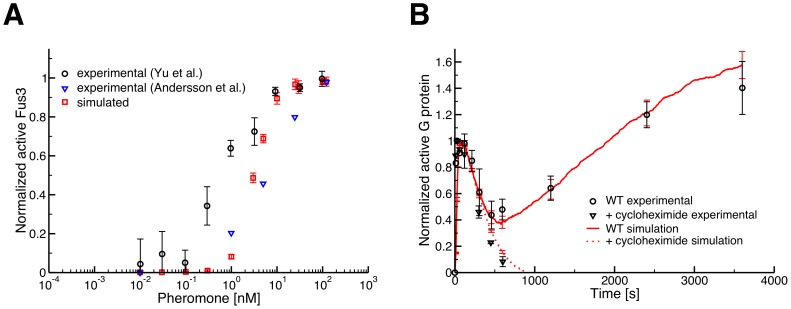
Experimental validation of the rule-based ensemble model. All error bars are 95% confidence intervals (simulated n = 10 where the simulations start from identical steady-state initial conditions). (A) Dose-response curves for Fus3 activation with respect to pheromone. The model displays similar behavior to that observed experimentally, although the experimental curves do not completely agree with one another [13], [14]. Also note that the level of noise observed in our simulations is equivalent to, if not less than, that observed *in vivo*. (B) G-protein activation time-course curves in response to 100 nM pheromone. An initial spike in activation, a subsequent decline and a long-term increase are seen upon pheromone stimulation in both wild-type FRET experiments [37] (black circles) and simulation (red solid line). Addition of cycloheximide in the experimental data (black triangles) indicates that the long-term increase in G-protein activation is due to pheromone-induced transcription [37].

**Figure 3:**
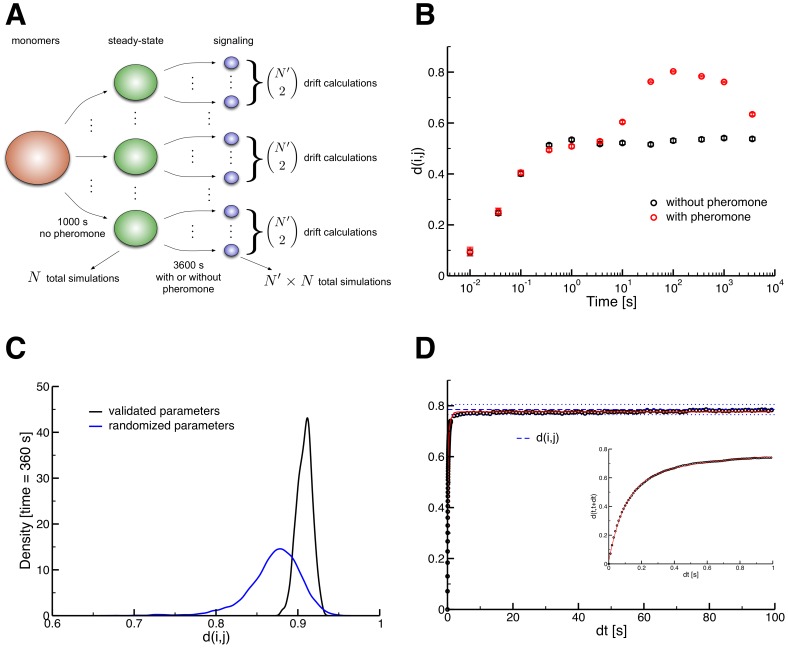
Characterization of heterogeneity among signaling species in the ensemble model. All error bars are 95% confidence intervals. (A) Visual depiction of our simulation method. An initial state was defined (red; all monomeric protein agents with one exception) and a number of trajectories were simulated to represent a set of untreated, homeostatic yeast cells (green). Pheromone was then added and each steady-state cell was simulated for a number of independent 1-hour trajectories (blue). Drift values were then calculated pairwise for all simulations that were derived from the same original homeostatic cell. The same procedure was also performed without adding pheromone as a control. (B) Average drift on a log time scale (n = 45). All simulations in this case (both with and without pheromone) start from *identical* steady-state initial conditions, so we have *d* = 0 at time *t* = 0. Simulations with pheromone (red) show a greater overall increase in drift as compared with simulations without pheromone (black). Peak levels of drift (∼0.8) occur around 100–300 seconds into simulations with pheromone. The ultimate decline in drift (*t* = 3600 s) is due to the presence of negative feedback in the cascade, returning the MAPK network to homeostasis. (C) Density of drift among scaffold-based complexes with randomized parameters (black, n = 9000) and the parameters that reproduce the experimental data (blue, n = 450). 1000 parameter sets were randomly generated and simulated, and we employed kernel density estimators to produce the density curves (Section 2.4 in Text S1). Though the mean drift values of the two distributions are significantly different (*p*<10^−5^, Section 2.4 in Text S1), the vast majority of drift values in the models with both randomized and fixed parameters are above 0.8, demonstrating the robustness of high drift in the model, regardless of rate parameters. (D) Autodrift occurs on two different timescales as indicated by statistical analysis of our double-exponential fit (n = 10, Section 3.3 in Text S1). Signaling events induce rapid divergence within one second (inset) from identical initial states within a particular simulation starting at the time of peak signal output (*t* = 300 s, Section 3.3 in Text S1) [47]. Also of note is the blue dashed line, which is the average drift between simulations at the 360 second time point (the blue dotted lines are the 95% confidence interval).

To create a dynamical model of this cascade, we constructed a set of rules for these interactions and other events (e.g. post-translational modification, protein synthesis and degradation, nucleotide transfer). The rules themselves, which follow mass-action kinetics, were primarily derived from two sources: an online model (http://yeastpheromonemodel.org) [40] and an ODE model [11], both of which are based on comprehensive literature searches (Section 1 in Supporting Information Text S1). In our initial model, if a reaction (e.g. efficient phosphorylation of Fus3 by Ste7) requires conditions that have been experimentally characterized (e.g. Ste7 also bound to Ste5), they are explicitly represented in the rule. We added no additional constraints to this model, in order to: (a) see if existing knowledge of these interactions is sufficient to produce realistic network dynamics (Fig. 2) and (b) characterize whether they result in machine- or ensemble-like character. The rule set, written in the Kappa rule-based modeling language [41], contains 232 rules, 18 protein and 8 gene agent types and is available as a separate supporting file (“ensemble.ka” in Protocol S1). This model displays considerable combinatorial complexity: even if we only focus on complexes containing the Ste5 scaffold, the system can generate over *3 billion* unique molecular structures (Section 3.5 in Text S1). We thus employed KaSim, an open source simulator for Kappa models, to consider the dynamics of the system without a reduction in its combinatorial complexity. Our general simulation strategy is described in detail in the Materials and Methods section and Section 2 in Text S1; a graphical schematic can be seen in Fig. 3A

### Parameterization of the model

The model described above has two types of parameters: initial copy numbers (i.e. concentrations) for each of the 18 protein agents and stochastic rate constants for each of the 232 rules. We obtained the initial conditions directly from experimental measurements of copy number in yeast cells [40], [42]. The stochastic rate constants were obtained from a combination of experimental data and parameter fitting. Briefly, 7% of the rate constant parameters in the model have been directly measured for yeast proteins, 68% were estimated from measurements on related proteins in other networks and 25% were completely unknown and thus given approximate values. In order to reproduce experimental observations with our model, we identified 111 rules that were likely to influence experimentally characterized trends and varied their rate constants.

We found that only 25 of these parameters had a strong impact on the dynamics of important observables in the model, and so we only modified those values during our fitting procedure. Of these 25, 22 had original estimates obtained from related proteins. In those cases, we restricted variation of the parameters to an increase or decrease of about one order of magnitude, to maintain similarity between the fitted value and the original estimate. Two of the remaining parameters had no available estimate, and so we restricted variations in those parameters to a biologically realistic range (a table with ranges for each type of parameter is available in Section 1.2 in Text S1). Finally, one parameter, the Gpa1 degradation rate, had been measured experimentally; we restricted variation in this parameter to a less than five fold change, a reasonable range given the inherent error in the experimental measurement [43]. Further details on how we identified and varied these parameters may be found in Section 1.2 in Text S1.

Since each simulation of this model requires over three hours of CPU time, we could not perform fits using standard techniques, nor could we employ statistical methods to understand the probabilistic structure of the parameter space [44], [45]. Therefore, we manually altered these 25 parameters (subject to the above constraints) and simulated the model with the updated rate parameters. We iteratively applied this procedure until the model successfully replicated the dose-response behavior of Fus3 with respect to pheromone (Fig. 2A) [13], [14], the temporal dynamics of G-protein activation (Fig. 2B) [37], and other experimental observations (Figs. S1, S2, S4, and S5 in Text S1). To test the robustness of our results to the particular simulation method, we translated our rules into the related BioNetGen Language (BNGL) and used the same parameters to simulate the model using the BNGL simulator NFsim [23]. The two software packages produced exactly the same dynamics for these rules (Figs. S4, S5 and Section 2.2 in Text S1). The BNGL version of the model is also available as a supporting file (“ensemble.bngl” in Protocol S1).

Given the large number of parameters in the model compared to the amount of data available for fitting, one should not construe the above results as implying this model represents a uniquely valid description of the system. Indeed, as we demonstrate below, even fairly different rule sets can provide (roughly) equivalent fits to this data; we thus cannot make any claims regarding the identifiability of the parameters or even the rule set itself [45], [46]. The point in this case is that it is possible to find some set of parameters that replicate the data, indicating that this model is at least consistent with available observations.

### Heterogeneity in signaling complexes

To determine if the model described above signals through ensembles, we implemented a pairwise comparison between the sets of complexes produced in two independent simulations *i* and *j*, using the Jaccard distance, which we refer to as “compositional drift” [16]:
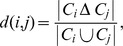
where *C_i_* represents the set of unique complexes in simulated cell *i*, Δ and 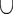 are the symmetric difference and union set operators, respectively, and |*X*| is the cardinality of set *X*. Given the complexes present in two simulated cells, drift is the number of complexes unique to *either* one cell or the other, divided by the total number of complexes in the union of the two cells. Drift can thus be interpreted as the probability that a complex found in one cell is not found in the other at a particular point in time. For example, *d* = 0 indicates identical sets of complexes, whereas *d* = 1 means the sets are pairwise disjoint. We only performed this comparison between multiple simulation replicates that started from *exactly the same* steady-state initial condition; thus *d* = 0 at *t* = 0 for all of our simulations (Fig. 3A; Sections 2.3 and 3.3 in Text S1). Note that this calculation takes into account any difference between complexes, whether the difference is in binding partners, phosphorylation states, or otherwise. Analysis of other potential criteria for differentiating complexes yielded similar results to those discussed below (Fig. S9 and Section 3.3 in Text S1).

We observed a marked increase of drift between simulations with pheromone (and thus signaling activity) as opposed to those without pheromone (Fig. 3B). At peak Fus3 signaling activity (*t* = 360 seconds), around 80% of *all* unique complexes were exclusive to one simulation or the other (Fig. 3B). Such small overlap indicates that individual cells utilize different sets of signaling complexes, consistent with the ensemble hypothesis [7], [16]. To confirm that this high level of drift is not an artifact of our chosen parameters, we generated over 1000 rule sets with randomized rate parameters (Section 2.4 in Text S1). In Fig. 3C we see the distributions of drift values among *scaffold-based* signaling species for both the validated model and models with randomized parameters at peak Fus3 signaling. Although the average random parameter set has somewhat lower drift than observed in our original parameter set, approximately 97% of the drift values from the models with randomized parameters were nonetheless greater than 0.8. The high level of drift among signaling species thus likely arises from the rules and interactions themselves rather than specific rate constants.

While the results in Fig. 3C indicate relatively high levels of heterogeneity at a particular time point, it could be that two different simulated cells utilize the same set of complexes, just at different times during signal transduction. We thus considered the differences between cells based on the union of all the unique complexes they sampled across the time points in our simulations (i.e. the points in Fig. 3B). We found that using the union of complexes across times only reduced absolute drift levels by about 10%, indicating a high degree of diversity between simulated cells across the entirety of the signaling dynamics (Fig. S10 in Text S1).

Our analysis of drift across time points raised the question of whether an individual simulation *i* maintains a specific set of complexes, or if the set changes over time. To answer this question, we used an alternative drift calculation, termed autodrift: *d_i_*(*t,t+*Δ*t*) instead of *d*(*i,j*). We found that simulated cells employ rapidly changing sets of complexes during peak signaling times in this model (Fig. 3D). Autodrift increased as a double exponential, with a longest time scale of approximately 0.5 s (Fig. 3D, inset, and Section 3.3 in Text S1). Indeed, within 5 seconds the difference between a cell and its past self achieves levels of drift similar to that observed between two completely independent cells in the population. This is consistent with observations from both modeling and experimental studies of epidermal growth factor signaling in mammals, where a diverse set of phosphorylated species forms rapidly during signaling [47], [48]. The rapid increase in drift also highlights the transient nature of the ensembles of complexes that are generated.

### Detailed analysis of signaling species

**Figure 4:**
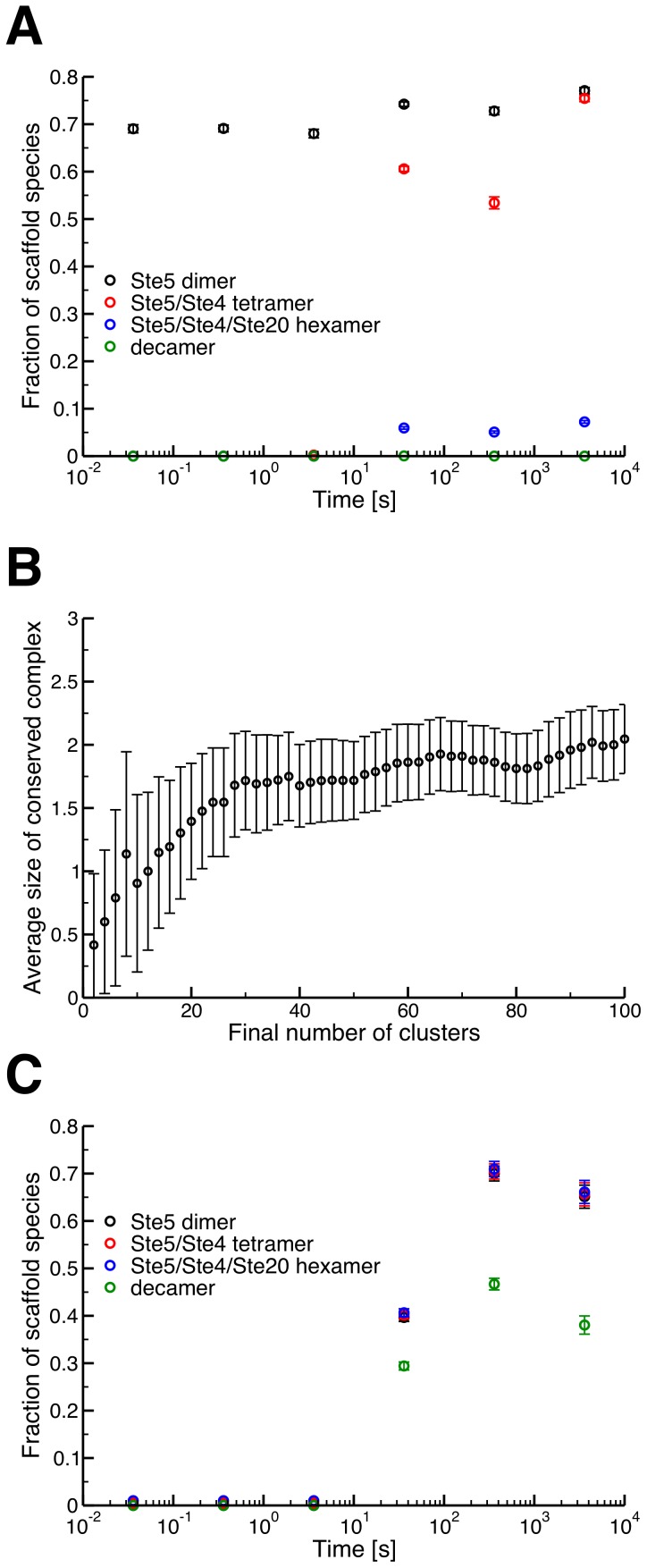
Structural analysis of complexes. (A) Structural conservation among scaffold-based signaling species in the ensemble model (n = 10). Ste5 dimers are present in 70% of species (black), however as we consider higher-order oligomers formed around this dimer, the fraction of species that contain these patterns drops sharply, with the fully bound Ste5-based decamer not seen at all. The standard depiction of the scaffold-based signaling complex (Fig. 1A) is thus unlikely to be observed in the ensemble model, if it occurs at all. Error bars are 95% confidence intervals. (B) The average size of the conserved protein complex as a function of the final number of clusters (i.e. the cutoff). At each cutoff, the average considers only those clusters with 10 or more members, to avoid contribution from very small collections of complexes. The average size does not exceed two, even considering up to 100 unique clusters. Error bars represent the 95% confidence interval of the mean. (C) Conservation of structure as in Fig. 4A, but in the machine model (n = 10). Here the decamer (signaling machine) is present in about 50% of species during peak signaling. The dimer, tetramer and hexamer patterns (black, red and blue, resp.) are present in identical fractions of the unique species, but are separated slightly in the graph for clarity.

It is possible that the putative ensembles in this case merely represent a set of highly similar (though technically distinct) signaling species that form around a large “core” signaling complex. We thus examined in detail the structures of the scaffold-based species at various time points in our simulations. If a core complex were present, we would expect to see substantial conservation of protein binding patterns (ignoring phosphorylation state) in the set of unique complexes. Though Ste5 dimers are present in ∼70% of species during peak signal throughput, conservation significantly declines as the binding pattern is expanded to include more proteins (Fig. 4A). In fact, not once did we find a Ste5 dimer bound to all its potential interaction partners, indicating that the complex used in the standard graphical depiction of this phosphorylation cascade is one that would very rarely, if ever, occur in simulations of this model (Fig. 1A) [8]–[14].

It is possible that complexes in the ensemble model still assemble around a consistent core structure, just not the traditional representation of a scaffold-based core signaling complex that we intuitively expect (Fig. 1A). Since there are over 3 billion possible scaffold signaling structures in this model, however, we could not search for this core by enumerating all possibilities and looking at conservation patterns as in Fig. 4A. We thus used a straightforward clustering analysis to search for an alternative core structure. The signaling species generated in our model were clustered on the basis of the structural similarity between complexes, represented in this case by the *graph edit distance metric*, which is simply the number of changes (or edits) that would be required to form one complex starting from another. This distance accounts for differences in the members of a complex (i.e. the removal of a protein from a complex increases the distance) as well as differences in phosphorylation state, etc. (Fig. S12 and Section 3.4 in Text S1).

We implemented a hierarchical clustering algorithm based on this distance. Briefly, the algorithm chooses a representative complex from each cluster, called the “clustroid,” which is the complex with the lowest average graph edit distance to all other complexes in its cluster (Section 3.4 in Text S1). At each level of the hierarchy, the algorithm combines the two clusters whose clustroids are most similar, that is those with the minimum graph edit distance (i.e. the minimum between-cluster distance, or MBCD). This algorithm is initialized with each complex in its own cluster (meaning the complex is its own clustroid) and continues until the original set of complexes is partitioned into a given number of clusters. This number, which we call the “cutoff,” is a free parameter and is relatively arbitrary in our case (Fig. S15 and Section 3.4 in Text S1), so we repeated the clustering algorithm with numerous different cutoff values. We calculated the size of the largest conserved structural pattern as a function of the cutoff value for each cluster that contained ten or more complexes. We found that, on average, this conserved pattern contained less than 2 proteins (Fig. 4B), indicating substantial dissimilarity among clustered proteins; cutoff values producing clusters with 4 or more proteins in the conserved subgraph were very rare (Fig. S15 in Text S1). These results, combined with the dissimilarity between clusters generated from independent simulations (Fig. S13 in Text S1) and the high levels of drift we observe (Fig. 3B–D), underscore the strong ensemble character of this model.

### Building a machine model based on a multi-subunit kinase

The findings described above indicate that heterogeneous ensembles of complexes can indeed transmit and process extracellular information with levels of noise comparable to those observed experimentally (Figs. 2–4). To understand if machine-like complexes could also produce reliable signaling behavior, we constructed an alternative model with the goal of assembling signaling machines, which we defined to be stable, multi-subunit kinases based around the scaffold Ste5 [1], [5], [9]. Specifically, the machine we focused on consists of a Ste5 dimer, with each scaffold protein bound to a Ste4–Ste20 dimer and two kinases, Ste11 and Ste7 (Fig. 1A). Upon assembly and activation, this decameric structure binds and phosphorylates Fus3 according to standard mass-action kinetics [9].

In contrast to the previous model, we were forced to introduce *a priori* assumptions (neither experimentally supported nor specifically refuted) in order to generate stable signaling machines. The simplest possible approach would be to create rules and rates that render the desired machine complex incredibly stable. The decamer, however, is essentially never generated in our original model's simulations (Fig. 4A), so a machine model based purely on increasing the stability of the desired complex is unlikely to actually produce such machines in high quantities reliably. As mentioned above, this fact resembles the Levinthal paradox in protein folding: no matter how stable the native state of a polypeptide chain may be, proteins would essentially never fold if they randomly searched for this state on an otherwise “flat” energy landscape [19], [20]. Alternatively, evidence suggests that molecular machines assemble hierarchically *in vivo* [49], and so we added specific rules that determine the order in which binding and phosphorylation could occur between the scaffold and its associated proteins (Fig. 1B, red arrows). This represents a hierarchical energy landscape (extending the analogy to protein folding), where each consecutive step builds toward the formation of a “native” signaling machine [19]. For example, in the machine model, binding of Ste11 to the scaffold can only take place if Ste5 has dimerized and each scaffold is bound to a Ste4–Ste20 dimer. Beyond these scaffold assembly rules, no other alterations were made to the model.

The resulting rule set is sufficiently complex that it is impossible to directly estimate the number of unique species that the machine model could form. We thus translated this model from Kappa into BNGL and used available BioNetGen tools to calculate the total number of species for this rule set [50]; as with our ensemble model, the Kappa and BNGL versions of the machine model are available as supporting files (“machine.ka” and “machine.bngl”, respectively, in Protocol S1). This analysis indicated that the machine model could only generate a total of 1106 possible scaffold-based structures, a decrease of over 6 orders of magnitude compared to the ensemble model (Section 3.5 in Text S1). The hierarchical assembly rules in this case thus drastically constrain the set of possible species that the model can sample.

### Differences between the machine and ensemble models

As with our original model, we subjected this alternative machine model to parameter variation and confirmed that it can reproduce experimental data (Figs. S6, S7, S8 and Sections 1.8 and 3.2 in Text S1). Although the dose-response and time-course trends of the machine and ensemble models are similar, they exhibit significantly different sets of signaling complexes. As expected, nearly half of all unique scaffold species in the machine model contained the decamer defined above (Fig. 4C), indicating wide conservation of the desired core signaling complex, in contrast to the complete lack of conservation observed in the ensemble model (Figs. 4A and 4B).

**Figure 5:**
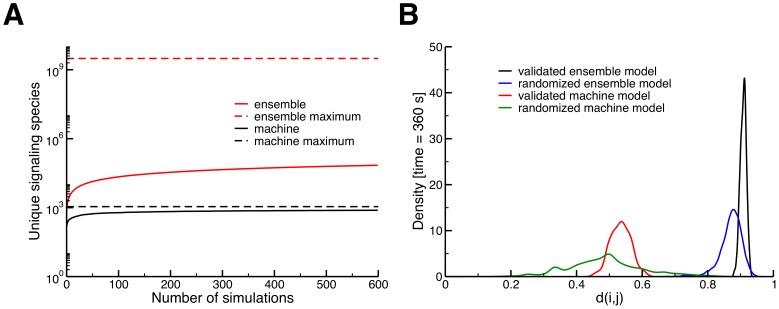
Comparison of notable characteristics in the machine and ensemble models. (A) Cumulative number of unique signaling complexes sampled by the machine and ensemble models (black and red, respectively) as a function of the number of independent simulations considered. We see that ensemble model generates a set of complexes approximately two orders of magnitude larger than the machine model over a range of 600 simulations. (B) Drift density among scaffold species in the validated machine model (n = 450) and 1000 machine models with randomized parameters (n = 7789) as compared to the data in Fig. 3C. A large difference between the machine and ensemble models can be seen, with significantly higher mean drift in both the validated ensemble model and the set of randomized ensemble models (both comparisons have a significance of *p*<10^−5^, see Section 2.4 in Text S1). It is also notable that the largest drift value from the machine model is much lower than the smallest from the set of ensemble models with randomized parameters. The remaining heterogeneity observed in the machine model can be attributed to the presence of assembly intermediates and regulatory interactions.

The set of species sampled in the machine model also differed dramatically from those produced by the ensemble model. As a gross estimate of this difference, we considered the *cumulative* number of unique scaffold-based species obtained by a set of simulations; that is, the total number of unique complexes that are found in a group of *N* simulated cells. In the machine model, this number rapidly approaches a maximum value as *N* increases, saturating at around 800 after considering only 100 simulations (Fig. 5A). The machine model thus samples about 70% of the 1106 possible scaffold complexes in a population of ∼100 cells. The behavior of the ensemble model is strikingly different, sampling a set of unique structures that is nearly two orders of magnitude greater than the machine model (approximately 70,000, Fig. 5A), and failing to saturate even after considering a population of 600 simulated cells. Although the total number of sampled species across these 600 cells is large, it is only 0.0022% of the 3 billion species the ensemble model could theoretically generate.

As one might expect given the results of Fig. 5A, we observed large differences in drift during peak signal output between the two models. On average, only 55% of unique scaffold complexes were exclusive to one of two simulations in the machine model, as opposed to 90% in the ensemble model (Fig. 5B). As with the ensemble model, we generated 1000 alternative machine models with randomized parameter sets to determine if the level of drift in this case was an artifact of the parameterization of the model. Though the distribution of drift values was fairly wide across these randomized models, in every case we observed considerably less drift than for the validated or randomized ensemble model (Fig. 5B). The rules underlying the machine model thus robustly produce dynamics that one might expect for well-established molecular machines like the ribosome or proteasome: a stable, heavily populated core structure with residual diversity arising from assembly intermediates and the association of substrates and/or regulatory factors.

### Evaluating experimental evidence for ensembles

**Figure 6:**
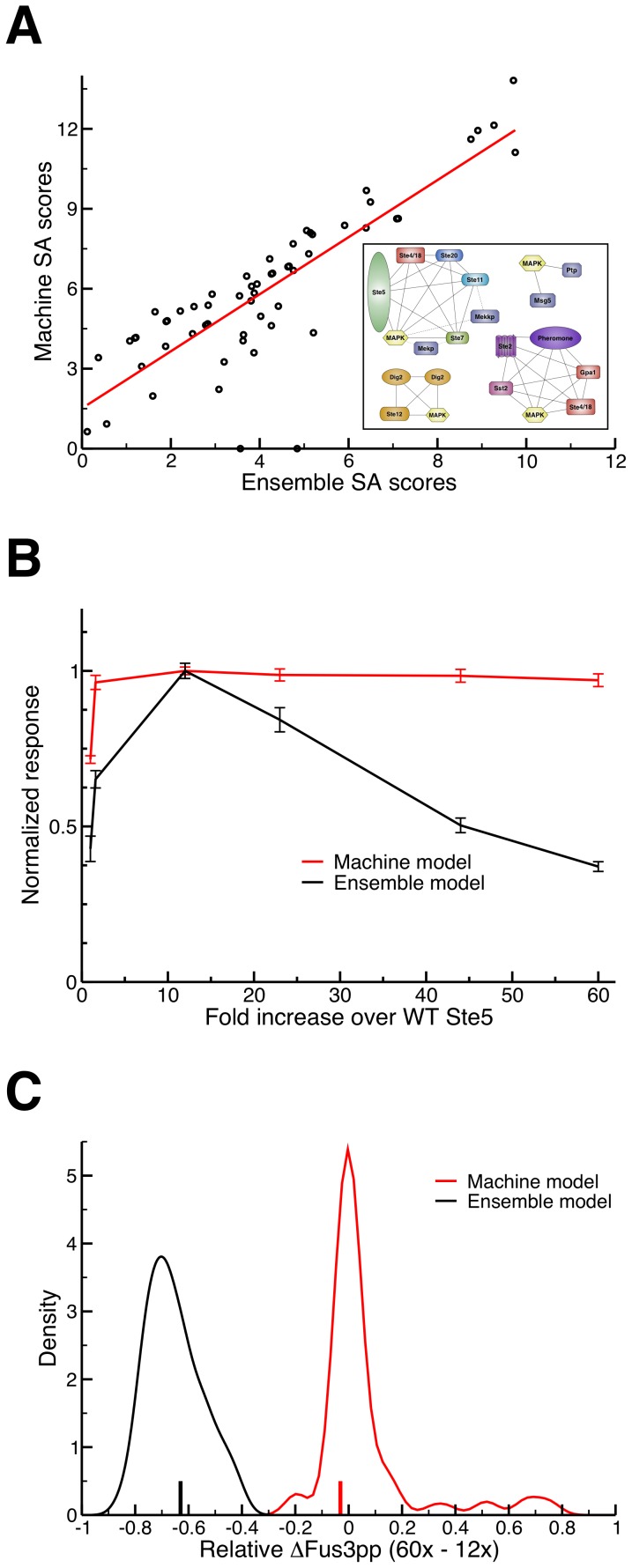
Indirect evidence for complex structure. (A) The socio-affinity (SA) scores [27] obtained from a computational TAP/MS experiment (averages over 10 simulations) where each point is the SA score between two specific proteins (e.g. Ste4 and Fus3) in both the ensemble (x-axis) and machine models (y-axis). The machine and ensemble models' scores exhibit a strong correlation and clustering them [28] resulted in highly similar “complexes” (inset, dashed lines exclusive to ensemble model, dotted to machine model), indicating that this method of characterizing *in vivo* complexes cannot distinguish between these two modes of signaling. (B) Overexpression of Ste5 in the machine (red) and ensemble (black) model results in different responses (n = 10). In addition to the phenotypic plasticity, we can see the clear presence of combinatorial inhibition [26] in the ensemble (but not machine) model as observed *in vivo* by Chapman and Asthagiri [12]. (C) We analyzed the robustness of combinatorial inhibition to variations in the parameters by considering 100 randomized ensemble models and 100 randomized machine models. In each case, we simulated the model with 12× and 60× the WT Ste5 concentration, and calculated the relative change in Fus3 activation, ΔFus3pp (60×–12×). The negative values for the distribution of ensemble models (black) indicates the robust presence of combinatorial inhibition, whereas the machine models (red) mostly have changes around zero with a few strongly positive outliers (which have been omitted for clarity). The difference in means between the two distributions is statistically significant (*p*<10^−5^, permutation test). Vertical bars on the *x*-axis indicate the relative ΔFus3pp (60×–12×) for the validated machine and ensemble models in red and black, respectively.

Since these two models can both reproduce general pheromone-dependent trends, one might ask if it is possible to differentiate machine- and ensemble-like signaling processes directly using available experimental techniques. The most natural approach would be tandem affinity purification in conjunction with mass spectrometry (TAP/MS), which is widely employed as a high-throughput assay for the discovery and analysis of protein complexes [27]. For example, Gavin *et al.* employed a “socio-affinity” (SA) index designed to extrapolate binary TAP/MS interaction data in order to discover novel “eukaryotic cellular machines” via clustering analysis [27]. To determine whether this technique could discern the nature of *in vivo* signaling complexes, we characterized the signaling species generated in both the ensemble and the machine models using the SA index [27]. There is a high correlation between the SA scores produced from our two models' sets of species (Fig. 6A); clustering these scores using the commonly employed MCL algorithm [27], [28], [51], [52] results in essentially the same set of complexes (Fig. 6A, inset).

This leads to the question of whether one could ever detect any functional differences between ensembles and machines in a signaling context. Previous work has established the presence of “combinatorial inhibition” [26] (akin to the “prozone” effect [53]) in this particular cascade; increased expression of the Ste5 scaffold leads to a maximal response, past which further overexpression leads to a decline in signal output [12], [40]. We found that the ensemble model reproduces this behavior, while the machine model does not (Fig. 6B). In the ensemble model, the eventual decrease in signal response arises because the high quantity of scaffold proteins lowers the probability of cascade components (say, Ste7 and Ste11) binding the *same* scaffold dimer [26], [53], and so the rate of signal propagation is drastically reduced. The hierarchical assembly rules in the machine model, however, reduce drift by ensuring scaffold dimers can only bind Ste7 after Ste11 is already bound. Beyond a certain minimal point, increasing Ste5 concentration has no effect, since the only potential scaffold binding partners for Ste7 are already bound to Ste11, and thus can propagate signal.

To test if the difference in Fig. 6B was robust to variations in the rate parameters, we simulated 100 randomized ensemble models and 100 randomized machine models with three values of Ste5 concentration: Wild Type (WT), 12 times WT (12×) and 60 times WT (60×). We used these simulations to calculate the relative change in peak Fus3 activation (ΔFus3pp) between two pairs of scaffold concentrations: WT to 12×, and 12× to 60×. The validated ensemble and machine models both exhibit a *positive* ΔFus3pp (12× – WT), corresponding to an increase in Fus3 activation (the peak in Fig. 6B); all the randomized ensemble models, and most of the randomized machine models, displayed this same behavior (Figs. S16, S17 and Section 3.7 in Text S1). In the ensemble model, increasing Ste5 to 60× WT concentration decreases response, yielding a negative ΔFus3pp (60×–12×), while the machine model exhibits an approximately constant response across these concentrations (Figs. 6B and C). The randomized ensemble models also universally showed a decrease in Fus3 activation from 12× to 60× Ste5 concentration, indicating that combinatorial inhibition is a robust feature of the ensemble model. The randomized machine models, however, had mostly *increases* in Fus3 activation between these two concentrations, and in no case did we observe a decrease as large as that observed for the ensemble models (Fig. 6C). The relative lack of combinatorial inhibition in the machine model is thus likely a feature of the rules themselves, rather than the specific parameters chosen.

It should be noted that the machine considered here is an *acyclic* complex; that is, there are no ring-like motifs in the protein interaction map for Ste5 (Fig. 1A) [53]–[57]. Previous modeling studies indicate that ring-like structures can assemble efficiently into well-defined quaternary structures, at least in certain parameter regimes [57]. Nonetheless, overexpression of a single subunit in a heteromeric ring causes a marked decrease in the concentration of the assembled machine, indicating that ring-like structures can simultaneously exhibit a machine-like character and combinatorial inhibition [53], [56], [57]. We leave full consideration of the interplay between robustness and topology in the evolution hierarchical assembly pathways to future work [56], [57].

## Discussion

The nature of the signaling complexes formed during signal transduction is foundational to how we conceptualize and understand information processing in cells. This is particularly true of scaffolds, whose primary function is to serve as a platform for the formation of multicomponent complexes that transmit signals [9]. The question of whether these complexes align more with the machine or ensemble paradigm is thus crucial for developing a principled picture of the roles scaffolds play. For instance, it has been posited that Ste5 acts to insulate pheromone signals from activating other, related MAP kinase cascades by sequestering active Ste11 in a pheromone-specific complex. This view is inconsistent with the ensembles we observe, however, since those involve appreciable concentrations of free, active Ste11; in contrast, the machine model produces essentially no active Ste11 molecules that are not bound to the scaffold. The capacity of Ste5 to fulfill the role of insulator in this pathway, or the need to posit other mechanisms such as cross-inhibition [8], [9], is thus directly related to the degree of ensemble character the network displays, a fact that highlights the central role that reasoning about quaternary structure plays in developing and evaluating hypothetical signaling mechanisms.

Our findings indicate that certain experimental methods, such as TAP/MS, are ill-equipped to directly resolve the structural details of signaling complexes in living cells. The difficulty in this case lies with the inherently *binary* nature of co-purification assays: they can tell us that two proteins interact in some way, but they tell us very little about the *global structural context* of the complexes in which those proteins are found. For example, in our computational TAP/MS experiment, we see that the overall pattern obtained by “tagging” each protein and recording its interaction partners is essentially the same for both the ensemble and machine models (Fig. 6A). This is due to the fact that, while the types of quaternary structures formed varies considerably between the two models (Fig. 4), the probability of observing any given pairwise association between two proteins is essentially the same. Our results thus indicate that it is problematic to construe clusters obtained from TAP/MS data as representing “cellular machines” in the classic sense [1], [27].

In contrast, experimental methods that can capture *ternary* or higher interactions (i.e. the simultaneous association of three or more distinct proteins) could be used to provide direct evidence for (or against) the hierarchical assembly of a signaling machine. For instance, in the machine model, Ste7 only binds Ste5 after Ste11 is already bound. Observation of Ste7-Ste5 association in the absence of Ste11 binding to Ste5 would thus provide evidence against the type of signaling machine considered here (Fig. 1B). Methods such as fragment complementation assays and fluorescence triple correlation spectroscopy could likely be used to probe these types of ternary association dynamics [29]–[31]. Alternatively, recent advances in single-molecule (super-resolution) microscopy (e.g. methods like PALM and STORM) could potentially track the assembly of machine- or ensemble-like signaling complexes [32]–[35].

While direct experimental tests of the ensemble hypothesis are currently lacking, inherent functional differences between machine and ensemble models can be used to provide indirect evidence for or against a particular paradigm. For instance, the hierarchical assembly rules that are required to reliably construct a functional scaffold-based signaling machine prevent our machine model from replicating the experimental observation of combinatorial inhibition (Fig. 6B) [9], [12], [26]. Our analysis of machine models with randomized parameters indicate that this is likely a general observation: in order to exhibit combinatorial inhibition, signaling networks must have the capacity to sample large sets of complexes, ultimately leading to ensemble behavior (Fig. 6C). Although more work is clearly needed to unambiguously resolve the question of machines vs. ensembles, our findings on combinatorial inhibition indicate that at least some degree of ensemble character is likely present in yeast pheromone signaling. It is also clear that the assembly pathways employed to form machines can have measurable, phenotypic consequences. As a result, even if one could determine experimentally the small set of machine-like complexes employed by some network, making a model that employs these machines, but ignores the mechanisms necessary to generate them [11], [21], may not accurately capture the response of the system to perturbations.

The presence of ensemble character in signaling also highlights a potential evolutionary trade-off between machines and ensembles in terms of their phenotypic plasticity. Considering again the analogy to protein folding, adopting a well defined, thermodynamically stable tertiary structure clearly enables the function of a vast array of protein domains (i.e. the general protein structure-function paradigm) [58]. In some cases, however, it has been posited that “intrinsically unstructured” (or unfolded) protein domains may have a distinct functional or evolutionary advantage: for instance, they may display greater interaction plasticity, binding specifically yet transiently with a large number of protein targets [58], [59]. Similarly, a protein with a robust, stable quaternary structure (i.e. a machine) [1], [7], [16] may be beneficial for the conservation of universal cellular tasks, like protein synthesis and degradation. In the case of signal transduction, however, ensembles may offer greater functional and evolutionary plasticity. For example, modifying Ste5 expression levels produces altered, but nonetheless functional, responses without the need to introduce complex, coordinated mutations to the reaction network's rule set (Fig. 6B) [36]. In this sense, both intrinsically disordered proteins and pleiomorphic ensembles may perform unique intracellular tasks precisely because they involve less well-ordered (tertiary or quaternary) structures. The ensemble character we observe could thus represent a form of weak regulatory linkage among genes, ultimately being responsible for the remarkable capacity of MAPK networks to exhibit different but meaningful phenotypes when they are re-wired, either through synthetic modifications or naturally over the course of evolution [9], [25], [36], [60].

Since machines do indeed form in some signaling networks (e.g. the apoptosome), there is likely a *spectrum* of structural specificity in the formation of complexes during signal transduction [1], [6], [7]. Indeed, one could modify the machine model presented here to include a finite probability of “off-pathway” binding events (e.g. some chance that Ste7 will bind Ste5 even if Ste11 is not already bound). Such models could exhibit intermediate levels of both drift and combinatorial inhibition (Figs. 5B and 6B); future work on this and related systems will be necessary to understand the particular functional and evolutionary consequences of a particular degree of ensemble-like character in any given system. Nonetheless, our work clearly demonstrates that large, heterogeneous ensembles can indeed reliably transmit and interpret extracellular information [7], [21], [22]. This hints at the existence of a new paradigm for molecular computation, one in which the evolution or engineering of “local” interaction rules allows for robust information processing in the absence of “global” order (i.e. a stable, multi-subunit signaling machine) [1], [5]. Understanding the consequences of this paradigm for robustness [61], plasticity [9], [36] and crosstalk [8] in signaling networks represents a crucial task for the emerging field of systems biology.

## Methods

### Simulation

The models in this work were simulated using KaSim, a stochastic simulator for rule-based models based on the Kappa language that is capable of stochastically sampling all possible species a given model can generate (Fig. 5B; Section 2.1 in Text S1) [23], [24]. The model is initialized with a set of (mostly) monomeric protein agents and simulated for 1000 seconds without pheromone to generate a steady-state population of *N* untreated “cells.” We treated the cells with pheromone, and generated a set of *N′* independent hour-long simulations from each steady-state starting cell. All of the complexes present in the simulation were recorded at logarithmically spaced time intervals. Compositional drift calculations were performed using these “snapshots;” we only performed this calculation between simulations that started from *exactly the same* initial conditions (Fig. 3A). We performed similar simulations to determine both dose-response and the time course trends. Further simulation details may be found in Section 2.3 in Text S1.

### Autodrift statistical fitting

Simulation data was fit to a set of exponential models using nonlinear least-squares regression. We found that a double exponential function was the best fit for the data upon analysis of the residuals and the statistical significance of the estimated model coefficients. The functional form of the model and the full statistical analysis can be found in Section 3.3 in Text S1.

### Complex classification and clustering

We focused primarily on the scaffold-based species for the analysis of structural conservation and subsequent clustering. These were defined as any complex that included a Ste5 agent or that could bind a free Ste5 agent. We created a vector notation to uniquely identify any scaffold-based complex to simplify the calculation of the graph edit distance between any two complexes (Figs. S11, S12, and Section 3.4 in Text S1). We then implemented the clustroid-based hierarchical clustering approach described in the main text. Other clustering criteria, such as standard single- and complete-linkage, gave similar results (Section 3.4 in Text S1).

### Socio-affinity scores and complex determination

We extracted all the binary interactions from the set of complexes generated by our simulations, artificially creating “bait” and “prey” association data. This computational version of the TAP/MS experimental procedure was used to generate the SA scores [27]. The MCL clustering algorithm [52] was then employed to generate the “functional modules” generally associated with such data sets [51]. More information on the SA score calculation and clustering algorithm can be found in Section 3.6 in Text S1.

## Supporting Information

Protocol S1 **Model files.** This zip file contains the ensemble and machine models in both Kappa and BNGL syntax: “ensemble.ka,” “ensemble.bngl,” “machine.ka,” and “machine.bngl.” Each file contains the agent declarations, initial conditions, rules, variables, and observables necessary for simulation. The Kappa models (“*.ka”) may be simulated using the freely available KaSim simulator (https://github.com/jkrivine/KaSim/). The BNGL models (“*.bngl”) must first be converted to XML format using BioNetGen prior to simulation with NFSim (http://emonet.biology.yale.edu/nfsim/).
(ZIP)

Text S1 **Supporting information.** This file contains the additional figures, tables, methods and explanations referenced in the main text.
(PDF)
