# Supplementary material for: Machines vs. Ensembles: Effective MAPK Signaling through Heterogeneous Sets of Protein Complexes
Source: PLoS Comput Biol. 2013 Oct 10;9(10):e1003278. doi: 10.1371/journal.pcbi.1003278 (PMC3794900; doi:10.1371/journal.pcbi.1003278)
Supplement: Text S1 — Supporting information. This file contains the additional figures, tables, methods and explanations referenced in the main text. (PDF) [file pcbi.1003278.s002.pdf]

Machines vs. Ensembles: Effective MAPK Signaling through Heterogeneous Sets of Protein Complexes

Supporting Information

Ryan Suderman[1] and Eric J. Deeds[1,2]

[1]Center for Bioinformatics, The University of Kansas, 2030 Becker Dr., Lawrence, KS 66047

[2]Department of Molecular Biosciences, The University of Kansas, 1200 Sunnyside Ave., Lawrence, KS 66047

Email: Eric J. Deeds - deeds@ku.edu;

Contents

1 Yeast Pheromone Signaling Model **3**

1.1 Initial conditions 3

1.2 Rate parameters 3

1.3 G-protein cycle 5

1.4 Ensemble MAPK cascade 7

1.5 MAPK cascade regulation 8

1.6 Nuclear interactions and regulation 10

1.7 Gene interactions and protein synthesis 11

1.8 Constructing the machine model 12

2 Model Simulation **14**

2.1 Kappa and KaSim 14

2.2 BioNetGen and NFsim 15

2.3 Simulation methods 15

2.4 Parameter randomization 16

3 Additional results **18**

3.1 Model validation 18

3.2 Machine model validation 19

3.3 Compositional drift 19
3.4 Species classification and clustering 23
3.5 Enumerating all possible species 26
3.6 Socio-affinity scoring 28
3.7 Robustness of combinatorial inhibition 31

References **33**

1 Yeast Pheromone Signaling Model

The model, rules and parameters used in our simulations were developed based on a number of sources, most notably the annotated online model found at http://www.yeastpheromonemodel.org that is written in the BioNetGen rule-based modeling language (BNGL)(1). Numerous additional rules (including those regarding the nuclear shuttling of Ste5) were derived from equations and mechanisms present in Shao *et al.*'s ODE model (2). Our final model, written in the Kappa language(3; 4; 5; 6; 7), has a total of 232 rules and all but one follow mass-action kinetics. The rules themselves are provided in an additional supplementary Kappa file.

1.1 Initial conditions

The initial conditions for our model (protein copy numbers) were derived from (8; 9) and the online model (OM) with the exception of the phosphatases for Ste11 and Ste7, which are unknown and estimated. All eight gene agents have a copy number of 1. Also it is important to note that the number of pheromone (i.e. α-factor) agents varied among simulations. Our dose-response curves clearly required different levels of pheromone stimulation, however for the drift calculations we used a concentration of 100 nM (10000 molecules).

Protein	Copy number
Pheromone	varies
Ste2	10000
Gpa1/Ste4 complex	10000
Gpa1 monomer	5000
Sst2	2500
Ste20	4200
Ste5	1680
Ste11	3500
Ste7	960
Fus3	20400
Kss1	20800
Msg5	38
Ptp	1270
Ste11-phosphatase (Mekkp)	1750
Ste7-phosphatase (Mekp)	1750
Dig1	3409
Dig2	1184
Ste12	1390

1.2 Rate parameters

In the following sections we will discuss the numerous rate parameters in our model of the yeast pheromone signaling system, and so, for clarity's sake, we have classified the parameters into three categories:

1. directly observed in yeast (D)
2. indirectly inferred from similar systems (e.g. ERK phosphorylation) or previously used in other models (I)
3. unknown and estimated (U)

In total, 17 (7%) of the rate parameters in the ensemble model were directly observed, 158 (68%) were inferred, and 57 (25%) were unknown and estimated.

In the main text, we mention that 111 parameters were identified as potentially influencing dynamical trends seen in experimental data. We varied these parameters and ultimately determined that 25 of them had a strong impact on the observed trends; these numbers are shown in red in the following subsections. Of these 25, 1 was directly observed, 22 were inferred, and 2 were unknown. We identified these parameters through trial and error, hypothesizing which parameters govern certain experimentally characterized trends (if no such hypothesis currently exists) and modifying them to better match said trends. For example, the Ste4 synthesis rate likely controls the long-term increase after the initial peak in the G protein activation time-course plot, as seen in the main text, Fig. 2B (10). We therefore altered this rate over numerous iterations until our simulations accurately reproduced the observed experimental trend. The subsequent table lists those parameters that were modified to fit experimental observation in addition to the specific trends they affect. It is important to note that, due to a lack of model identifiability, there may be other parameters that alter the experimental trends in question; we chose these parameters simply due to their large relative influence on the dynamics of observables.

**Rate Parameter**	**Trend**	**Cat.**
Sst2/Ste2 assoc.	controls slope of decline in G protein activation curve following the initial peak	I
Sst2/Ste2 dissoc.	same as above	I
GTP hydrolysis	controls the time of the initial G protein activation peak	I
Gpa1 degradation	controls G protein levels and the relative levels of G protein activation	D
Ste4 degradation	same as above	I
Gpa1/Ste4 dimer deg.	same as above	I
Ste4/Ste20 dissoc.	determines the time course of Ste4-Ste20 binding	I
Ste4/Ste5 dissoc. (2 rates)	controls membrane localization of Ste5	I
Fus3 phos. (4 rates)	controls time and absolute value of Fus3 activation	I
Ste11 degradation	controls Ste11 levels and thus active Fus3 levels by extension (also involved with negative feedback and thus dose-response (DR) alignment)	I
Fus3/Msg5 dissoc. (4 rates)	controls peak Fus3 activation and DR trends	I
Fus3/Ptp dissoc. (2 rates)	same as above	I

Rate Parameter	Trend	Cat.
Fus3 dephos. by Ptp	controls peak Fus3 activation and DR trends	I
Ste12/Gpa1_gene assoc.	controls rate of Gpa1 synthesis (G protein activation) and thus the relative level of active G protein compared to the initial peak	U
Gpa1 synthesis	same as above	I
Ste12/Ste4_gene assoc.	same as above	U
Ste4 synthesis	same as above	I

In order to maintain a biologically realistic model, our modifications to these rates were confined to reasonable limits. We allowed variation of approximately one order of magnitude for parameters inferred from related systems and completely unknown parameters were estimated according to the following table:

Reaction Type	Parameter Range
K_D for cytosolic protein-protein interactions	10^2 nM
K_D for nuclear-localized protein-protein interactions	$10^1 - 10^3$ nM
k_{cat} for catalysis reactions	$10^{-1} - 10^1$ s^{-1}
k_{deg} for degradation reactions	$10^{-4} - 10^{-2}$ s^{-1}
k_{synth} for synthesis reactions	$10^{-1} - 10^1$ s^{-1}

In subsections 1.3 - 1.8 there are tables of the associated rate parameters used in the model and their sources (OM for online model). The leftmost column contains the interaction or reaction. These are condensed descriptions of the actual rules, which are explicitly defined in the Kappa rule file. As such, there may be numerous rates for a particular interaction or reaction indicating that the rate differs in specific contexts (e.g. binding partners, phosphorylation states). The center-left column contains the rate parameter(s) and the center-right column mentions the model or other source from which it was derived. The parameter's category (D, I or U) is seen in the rightmost column. Note that as these are stochastic rates; the parameters for bimolecular reactions thus depend on the volume used for the yeast cell(9; 11), which may not be identical between models.

1.3 G-protein cycle

The initial events of the pheromone response network involve the extracellular pheromone binding to the G-protein coupled receptor, Ste2. Since we are implementing a stochastic model and our rates require a specific volume, we define our extracellular volume to be $V_{ext} = 166$ fL and our intracellular volume to be $V_{int} = 19.3$ fL (12). Briefly, the G-protein cycle passes the extracellular signal (the presence of pheromone) to the MAPK cascade via a nucleotide exchange mechanism. Our understanding of this process in yeast comes from (2; 10). Upon activation of the G-protein coupled receptor (Ste2), the α subunit of the heterotrimeric G-protein (Gpa1), bound to Ste2,

exchanges its bound GDP for GTP, thereby inducing dissociation from the $\beta\gamma$ complex (Ste4-Ste18, subsequently referred to just as Ste4). This allows Ste4, which is tethered to the membrane via Ste18 (implicit in our model) to recruit Ste5 and induce the MAPK cascade (1.4,1.8). The GTPase-Activating Protein (GAP), Sst2, is able to bind Ste2, and the resulting colocalization of Sst2 and Gpa1 via Ste2 catalyzes GTP hydrolysis. Sst2 thus acts as a negative regulator, and enables Gpa1 to rebind Ste4 (10).

Note that numerous association rates among binary interactions in the G-protein cycle (Ste2/Gpa1 binding) are significantly higher than those later in the cascade (e.g. Ste5/Ste7 binding). This is due to the membrane association and localization of certain proteins (e.g. Ste4) and results in a higher apparent association rate (derived in the online model).

**Interaction or Reaction**	**Rate parameter(s)**	**Source**	**Cat.**
Pheromone/Ste2 interaction	3×10^{-5} molec^{-1}s^{-1}<br>0.015 s^{-1}	OM,(10)<br>OM,(10)	D<br>D
Ste2/Gpa1 assoc.	0.001725 molec^{-1}s^{-1}	derived	U
Ste2/Gpa dissoc.	0.15 s^{-1}<br>0.03 s^{-1}	OM<br>N/A	I<br>U
Gpa1/Ste4 assoc.	0.001725 molec^{-1}s^{-1}<br>8.595×10^{-8} molec^{-1}s^{-1}	derived<br>derived	I<br>I
Gpa1/Ste4 dissoc.	7.5 s^{-1}<br>1.5 s^{-1}	N/A<br>N/A	U<br>U
GDP $\rightarrow$ GTP	0.15 s^{-1}	(10)	D
Sst2/Ste2 interaction	8.595×10^{-4} molec^{-1}s^{-1}<br>0.15 s^{-1}	derived<br>derived	I<br>I
GTP $\rightarrow$ GDP	0.015 s^{-1}<br>0.015 s^{-1}<br>1.5 s^{-1}<br>1.5 s^{-1}	OM<br>OM<br>OM<br>OM	I<br>I<br>I<br>I
Sst2/MAPK assoc. (2 MAPKs)	8.595×10^{-5} molec^{-1}s^{-1}	derived	I (2)
Sst2/MAPK dissoc.	1.5 s^{-1}<br>0.75 s^{-1}<br>0.75 s^{-1}<br>0.375 s^{-1}	OM<br>OM<br>OM<br>OM	I (2)<br>I (2)<br>I (2)<br>I (2)
Sst2 phosphorylation	1.5 s^{-1}	OM	I
Sst2 dephos.	0.00087 s^{-1}	(2)	I
Ste2 endocytosis	0.0004 s^{-1}<br>0.003 s^{-1}	(10)<br>(10)	D<br>D

Interaction or Reaction	Rate parameter(s)	Source	Cat.
G-protein degradation	4.95×10^{-5} s^{-1}	OM	D
	4.95×10^{-5} s^{-1}	OM	I
	3.3×10^{-5} s^{-1}	OM	I
Sst2 degradation	0.0004 s^{-1}	OM	D
	0.0006 s^{-1}	OM	D

1.4 Ensemble MAPK cascade

Upon dissociation from Gpa1, Ste4 can engage in a number of different interactions. It can of course rebind Gpa1, but it can also bind the p21-activated kinase (PAK) Ste20 and recruit the scaffold protein, Ste5, to initiate the MAPK cascade (13). Ste5 in turn must bind Ste11, a MAPKKK, simultaneously with a Ste4 bound to a Ste20 and form a 4-member complex in order for the Ste20 to phosphorylate Ste11. Though this complex is required for signal transduction, no experimental evidence suggests any particular binding order for creation of this tetramer. Upon Ste5 dimerization, Ste11 can then cross-phosphorylate the MAPKK, Ste7, on the opposite Ste5(14). Active Ste7 bound to Ste5 can then phosphorylate the MAPK, Fus3 (13). As mentioned in the main text, only those dependencies explicitly demonstrated experimentally are implemented in our ensemble model (e.g. Ste5 need not necessarily be bound to Ste4 for Ste7 to phosphorylate Fus3). Note that interactions/reactions mentioning "MAPK" mean that the particular event could involve either Fus3 *or* Kss1 (a Fus3 paralog).

Interaction or Reaction	Rate parameter(s)	Source	Cat.
Ste4/Ste20 interaction	8.595×10^{-5} molec^{-1}s^{-1}	derived	I
	0.8 s^{-1}	derived	I
Ste4/Ste5 assoc.	0.001725 molec^{-1}s^{-1}	derived	I
	8.595×10^{-5} molec^{-1}s^{-1}	derived	I
	8.595×10^{-5} molec^{-1}s^{-1}	derived	I
Ste4/Ste5 dissoc.	0.2 s^{-1}	derived	I
	0.02 s^{-1}	derived	I
Ste5/Ste5 dimerization	8.595×10^{-5} molec^{-1}s^{-1}	derived	I
	8.595×10^{-5} molec^{-1}s^{-1}	derived	I
	0.001725 molec^{-1}s^{-1}	derived	I
Ste5/Ste5 dissoc.	0.075 s^{-1}	N/A	U
	0.0075 s^{-1}	N/A	U
	0.0005 s^{-1}	N/A	U
Ste11/Ste5 interaction	8.595×10^{-5} molec^{-1}s^{-1}	OM	D
	0.1605 s^{-1}	OM	D
Ste11 phosphorylation	0.5 s^{-1}	(2)	I
	0.5 s^{-1}	(2)	I
	0.5 s^{-1}	(2)	I

Interaction or Reaction	Rate parameter(s)	Source	Cat.
Ste5/Ste7 interaction	8.595×10^{-5} molec^{-1}s^{-1}<br>8.595×10^{-7} molec^{-1}s^{-1}<br>0.153 s^{-1}	OM,(15)<br>OM,(15)<br>OM,(15)	D<br>I<br>D
Ste7 phosphorylation	0.495 s^{-1} (12 rules)	OM	I (12)
MAPK/Ste7 interaction	4.35×10^{-6} molec^{-1}s^{-1}<br>0.0075 s^{-1}	OM<br>OM	D<br>D
Fus3 phosphorylation	7.5 s^{-1} (4 rules)	OM	I (4)
Kss1 phosphorylation	1.5 s^{-1} (4 rules)	OM	I (4)
MAPK autophosphorylation	4×10^{-4} s^{-1}	N/A	U (2)
Ste5/Fus4 interaction	8.595×10^{-5} molec^{-1}s^{-1}<br>1.425 s^{-1}	OM<br>OM	D<br>D

1.5 MAPK cascade regulation

Our model includes two MAPK phosphatase agents, Msg5 and Ptp (the latter of which represents two *in vivo* phosphatases, Ptp2 and Ptp3) that dephosphorylate Fus3(2). We also included individual phosphatases for Ste11 and Ste7, though the proteins which play this role *in vivo* remain to be experimentally characterized(2). Also, this model employs a number of feedback mechanisms(2). Active Fus3 can phosphorylate Ste11 (on a domain distinct from its activation domain) and Sst2, tagging them for degradation, which is modeled implicitly through faster degradation rates. Additionally, Ste7 is proposed to be hyperphosphorylated upon activating Fus3, tagging it for ubiquitination and subsequent degradation(16). This is implemented with a degradation rule that is dependent on Ste7's phosphorylation state.

Interaction or Reaction	Rate parameter(s)	Source	Cat.
Ste11 autodephos.	0.00087 (4 rules)	(2)	I (4)
Ste7 autodephos.	0.00087 (2 rules)	(2)	I (2)
Ste11/MAPK interaction	8.595×10^{-5} molec^{-1}s^{-1}<br>1.5 s^{-1}<br>0.75 s^{-1}<br>0.75 s^{-1}<br>0.375 s^{-1}	OM<br>derived<br>derived<br>derived<br>derived	I (2)<br>I (2)<br>I (2)<br>I (2)<br>I (2)
Ste11 phos. (by both MAPKs)	1.5 s^{-1}	OM	I (2)
Ste11 degradation	0.00075	OM	I
Ste5 phos.	1.5 s^{-1}	OM	I
Ste5 autodephos.	0.0087	(2)	I
MAPK autodephos.	0.00087 (4 rules)	(2)	I (4)

Interaction or Reaction	Rate parameter(s)	Source	Cat.
MAPK/Msg5 assoc.	8.595×10^{-5} molec^{-1}s^{-1}	derived	I
Fus3/Msg5 dissoc.	7.5 s^{-1}<br>3 s^{-1}<br>3 s^{-1}<br>3 s^{-1}	OM<br>OM<br>OM<br>OM	I<br>I<br>I<br>I
Kss1/Msg5 dissoc.	1.2 s^{-1}<br>0.12 s^{-1}<br>0.12 s^{-1}<br>0.12 s^{-1}	OM<br>OM<br>OM<br>OM	I<br>I<br>I<br>I
MAPK dephos. (Msg5, both MAPKs)	0.12 s^{-1}<br>0.12 s^{-1}	OM<br>OM	I (2)<br>I (2)
MAPK/Ptp assoc.	8.595×10^{-5} molec^{-1}s^{-1}	derived	I (2)
Fus3/Ptp dissoc.	1.5 s^{-1}<br>0.3 s^{-1}	OM<br>OM	I<br>I
Kss1/Ptp dissoc.	0.15 s^{-1}<br>0.03 s^{-1}	OM<br>OM	I<br>I
Fus3 dephos. by Ptp	1.2 s^{-1}	(2)	I
Kss1 dephos. by Ptp	0.12 s^{-1}	(2)	I
Fus3 degradation	0.0002 s^{-1}	N/A	U
Msg5 degradation	0.0008 s^{-1}	N/A	U
Ste7 hyperphos.	0.495 s^{-1}	N/A	U
Ste7 degradation	0.002 s^{-1}	(2)	I
Ste(11,7)/Phosphatase interaction	7.155×10^{-5} molec^{-1}s^{-1}<br>0.6 s^{-1}	N/A<br>N/A	U (2)<br>U (2)
Ste(11,7) dephos.	0.25 s^{-1} (5 rules)	N/A	U (5)
Ste5 nuclear import	0.5 s^{-1}	(2)	I

Ste5 also plays a role in regulating signal throughput, via feedback phosphorylation by Fus3 as well as by its presence in the cytoplasm. Ste5 is shuttled out of the nucleus, where it is normally sequestered, upon pheromone stimulation, though the precise mechanisms are unknown(17). Therefore, we implement this export rate (S_{exp}) as an equation where G_f is the number of Gpa1's that are not bound to a Ste4:

$$S_{exp} = 0.3 \cdot \left(\frac{G_f}{G_f + 2500} \right).$$

This is the only rule that does not follow the law of mass action and was obtained from Shao *et al.*'s export rate equation. The only difference in this case is that our rate does not have a basal value of 0.0003 when $G_f = 0$.

1.6 Nuclear interactions and regulation

Once Fus3 is activated, it translocates to the nucleus where it plays an active role in regulating genes associated with mating. Specifically, it inhibits Dig1 and Dig2 activity via phosphorylation. These two proteins, when not phosphorylated, bind to the transcription factor, Ste12, and prevent it from activating mating-related genes (13).

**Interaction or Reaction**	**Rate parameter(s)**	**Source**	**Cat.**
Dig1/Ste12 interaction	8.595×10^{-4} molec^{-1}s^{-1}	derived	I
	30 s^{-1}	N/A	U
	3 s^{-1}	N/A	U
	3 s^{-1}	N/A	U
	0.003 s^{-1}	N/A	U
Dig2/Ste12 interaction	8.595×10^{-4} molec^{-1}s^{-1}	derived	I
	30 s^{-1}	OM	U
	3 s^{-1}	OM	U
Fus3/Ste12 interaction	8.595×10^{-5} molec^{-1}s^{-1}	derived	I
	3 s^{-1}	OM	U
	15 s^{-1}	OM	U
	15 s^{-1}	OM	U
	75 s^{-1}	OM	U
	0.3 s^{-1}	OM	U
	1.5 s^{-1}	OM	U
	1.5 s^{-1}	OM	U
	7.5 s^{-1}	OM	U
Kss1/Ste12 interaction	8.595×10^{-5} molec^{-1}s^{-1}	derived	I
	0.75 s^{-1}	OM	U
	3.75 s^{-1}	OM	U
	3.75 s^{-1}	OM	U
	18.75 s^{-1}	OM	U
	0.075 s^{-1}	OM	U
	0.375 s^{-1}	OM	U
	0.375 s^{-1}	OM	U
	1.5 s^{-1}	OM	U
Fus3/Dig1 interaction	8.595×10^{-5} molec^{-1}s^{-1}	derived	I
	4.5 s^{-1}	OM	I
	2.25 s^{-1}	OM	I
	2.25 s^{-1}	OM	I
	1.125 s^{-1}	OM	I

Interaction or Reaction	Rate parameter(s)	Source	Cat.
Kss1/Dig1 interaction	8.595×10^{-6} molec^{-1}s^{-1}	derived	I
	7.5 s^{-1}	OM	I
	3.75 s^{-1}	OM	I
	3.75 s^{-1}	OM	I
	1.875 s^{-1}	OM	I
Fus3/Dig2 interaction	8.595×10^{-5} molec^{-1}s^{-1}	derived	I
	1.5 s^{-1}	OM	I
	0.75 s^{-1}	OM	I
	0.75 s^{-1}	OM	I
	0.375 s^{-1}	OM	I
Kss1/Dig2 interaction	8.595×10^{-5} molec^{-1}s^{-1}	derived	I
	2.55 s^{-1} OM	I	
	1.275 s^{-1} OM	I	
	1.275 s^{-1} OM	I	
	0.645 s^{-1}	OM	I
Dig phos. (2 MAPKs, 2 Dig proteins)	1.5 s^{-1}	OM	I (4)
Dig dephos. (2 Dig proteins)	0.00087 s^{-1}	(2)	I (2)
Dig2 degradation	0.0002 s^{-1}	N/A	U

1.7 Gene interactions and protein synthesis

Upon pheromone stimulation, a number of genes in the mating cascade itself are expressed at higher levels (*STE2, SST2, GPA1, STE4, FUS3,* etc.), providing a measure of feedback (18). Basal transcription of certain proteins is also present in the model.

Interaction or Reaction	Rate parameter(s)	Source	Cat.
Ste12/Ste2 gene interaction	2.145×10^{-5} molec^{-1}s^{-1}	derived	U
	0.03 s^{-1}	derived	U
Ste2 synthesis	3 s^{-1}	OM	I
	12 s^{-1}	(10)	D
Ste12/Gpa1 gene interaction	2.145×10^{-3} molec^{-1}s^{-1}	derived	U
	0.03 s^{-1}	derived	U
Gpa1 synthesis	27 s^{-1}	OM	I
Ste12/Ste4 gene interaction	2.145×10^{-4} molec^{-1}s^{-1}	derived	U
	0.03 s^{-1}	derived	U
G-protein basal synthesis	0.5 s^{-1}	OM	I
Ste4 synthesis	18 s^{-1}	OM	I

Interaction or Reaction	Rate parameter(s)	Source	Cat.
Ste12/Sst2 gene interaction	2.145×10^{-5} molec^{-1}s^{-1}<br>0.03 s^{-1}	derived<br>derived	U<br>U
Sst2 synthesis	0.78 s^{-1}<br>1.5 s^{-1}	OM<br>OM	I<br>I
Ste12/Fus3 gene interaction	2.145×10^{-5} molec^{-1}s^{-1}<br>0.03 s^{-1}	derived<br>derived	U<br>U
Fus3 synthesis	4 s^{-1}<br>15 s^{-1}	OM<br>OM	I<br>I
Ste12/Msg5 gene interaction	2.145×10^{-5} molec^{-1}s^{-1}<br>0.03 s^{-1}	derived<br>derived	U<br>U
Msg5 synthesis	0.08 s^{-1}<br>0.63 s^{-1}	OM<br>OM	I<br>I
Ste12/Dig2 gene interaction	2.145×10^{-5} molec^{-1}s^{-1}<br>0.03 s^{-1}	derived<br>derived	U<br>U
Dig2 synthesis	0.24 s^{-1}<br>0.45 s^{-1}	OM<br>OM	I<br>I
Ste12/Ste12 gene interaction	2.145×10^{-5} molec^{-1}s^{-1}<br>0.03 s^{-1}	derived<br>derived	U<br>U
Ste12 synthesis	0.45 s^{-1}	OM	I,I

1.8 Constructing the machine model

A number of rules were specifically designed to create a model that could assemble signaling machines. The interactions were arranged in a hierarchy to mimic the assembly of experimentally characterized machines(19). Specifically, in the machine model Ste5 can only bind a Ste4 that is bound to a Ste20. In order to proceed to the MAPK cascade, a dimer of the Ste5-Ste4-Ste20 trimers must form. This hexamer can assemble in two ways: a trimer can bind another trimer, or a trimer can bind a free Ste5 and subsequently bind a Ste4-Ste20 dimer. Then Ste11 can bind Ste5, and in order for Ste7 to bind Ste5, both Ste5 proteins must be bound to a Ste11 (forming an octomer). Only after both Ste7 proteins have bound to assemble the full decamer structure can phosphorylation occur. Once all four kinases are fully phosphorylated, the machine binds and phosphorylates Fus3 as a multi-subunit kinase.

Dissociation of the machine can proceed in two ways. The first is a disassembly pathway which is essentially the inverse of the assembly pathway. However, once the decamer is fully assembled, our rates are implemented in a way that reflects the inherent stability of a machine's quaternary structure(19). Thus we adapted the Ste7 hyperphosphorylation mechanism (that is present in the ensemble model, 1.5) to promote rapid dissociation of the signaling machine into its constituent monomers, and mimic the ability of Fus3 to induce negative feedback(2).

The following table of rates are those involved with rules that mechanistically differ from the ensemble model. Since these interactions were invented in the absence of any experimental evidence, the rates, in addition to the mechanisms, are hypothetical and were implemented in order to replicate experimental time-course and dose-response trends. The association rates were designed to be as similar as possible to those present in the ensemble model, however a few required manipulation to match experimental data. Varying the equation governing Ste5 nuclear export (S_{exp}^{mach}) was the primary means of replicating the dose-response curve. In particular it was altered to be more sensitive to the amount of free Gpa1 in the form of a Hill function:

$$S_{exp}^{mach} = 0.3 \cdot \left(\frac{{G_f}^4}{{G_f}^4 + 12000^4} \right).$$

Novel machine model events

Interaction or Reaction	Rate parameter(s)	Source	Cat.
Ste5/Ste4-20 interaction	8.595×10^{-5} molec^{-1}s^{-1}<br>0.2 s^{-1}	N/A<br>N/A	U<br>U
Ste5-4-20/Ste5 interaction	8.595×10^{-5} molec^{-1}s^{-1}<br>0.2 s^{-1}	N/A<br>N/A	U<br>U
Ste5-4-20-5/Ste4-20 interaction	0.001725 molec^{-1}s^{-1}<br>0.0005 s^{-1}	N/A<br>N/A	U<br>U
Ste5-4-20/Ste5-4-20 interaction	8.625×10^{-4} molec^{-1}s^{-1}<br>0.0005 s^{-1}	N/A<br>N/A	U<br>U
hexamer/Ste11 interaction	8.595×10^{-4} molec^{-1}s^{-1}<br>0.01605 s^{-1}	N/A<br>N/A	U<br>U
octomer/Ste7 interaction	8.595×10^{-4} molec^{-1}s^{-1}<br>8.595×10^{-6} molec^{-1}s^{-1}<br>0.0153 s^{-1}<br>1.53×10^{-6} s^{-1}	N/A<br>N/A<br>N/A<br>N/A	U<br>U<br>U<br>U
decamer activation (phos.)	0.1 s^{-1} (5 rules)	N/A	U
MAPK/decamer interaction	8.595×10^{-5} molec^{-1}s^{-1}<br>0.075 s^{-1}	N/A<br>N/A	U<br>U
MAPK dissoc.	10 s^{-1} (2 MAPKs)	N/A	U (2)
MAPK phos.	0.1 s^{-1} (2 rules, 2 MAPKs)	OM	I (2,2)
Ste7 hyperphos.	8.595×10^{-5} molec^{-1}s^{-1}<br>0.1 s^{-1}<br>0.1 s^{-1}<br>1 s^{-1}	N/A<br>N/A<br>N/A<br>N/A	U<br>U<br>U<br>U
Ste7 dephos. (alternate site)	0.0087	(2)	I

The subsequent table are those reaction events that have identical mechanisms but different rate constants between the two models.

Identical reactions with different rates

Interaction or Reaction	Ensemble model rate	Machine model rate
Sst2/Ste2 dissociation	0.15 s^{-1}	0.015 s^{-1}
Ste4/Ste20 interaction	8.595×10^{-5} $molec^{-1}s^{-1}$<br>0.8 s^{-1}	8.595×10^{-4} $molec^{-1}s^{-1}$<br>0.08 s^{-1}
Ste5/Fus3 dissoc.	1.425 s^{-1}	1 s^{-1}
Ste11/7 autodephos.	0.00087 s^{-1} (6 rules)	0.0087 s^{-1} (6 rules)
Msg5/Kss1 dissoc.	1.2 s^{-1}<br>0.12 s^{-1} (3 rules)	0.12 s^{-1}<br>0.012 s^{-1} (3 rules)
Fus3/Dig1 assoc.	8.595×10^{-5} $molec^{-1}s^{-1}$	8.595×10^{-4} $molec^{-1}s^{-1}$
Kss1/Dig1 assoc.	8.595×10^{-6} $molec^{-1}s^{-1}$	8.595×10^{-5} $molec^{-1}s^{-1}$
Dig phos. (2 MAPK, 2 Dig proteins)	1.5 s^{-1} (4 rules)	15 s^{-1} (4 rules)
Dig dephos. (2 Dig proteins)	0.00087 s^{-1} (2 rules)	0.001 s^{-1} (2 rules)
Ste5 phos.	1.5 s^{-1}	1 s^{-1}
Ste(11,7) dephos.	0.25 s^{-1} (5 rules)	1 s^{-1} (5 rules)

2 Model Simulation

Our model was simulated using rule-based techniques, in particular the Kappa rule-based modeling language(3; 4; 5; 6; 7) and its associated simulator, KaSim(20). As mentioned in the main text, these methods allow us to incorporate and investigate the influence of combinatorial complexity in our modeling without needing to explicitly enumerate all the possible species that our model can generate(11; 20). This section describes how we implemented and simulated our model as well as our method for randomizing our parameters in order to characterize the robustness of drift (3.3) in our simulations.

2.1 Kappa and KaSim

Kappa and related languages employ rules to define reaction network dynamics. Stochastic simulation of these rules using KaSim involves an adapted version of the Gillespie algorithm(21). Briefly, a rule has a left hand side (LHS), a right hand side (RHS) and an associated stochastic rate constant. Both the LHS and RHS are site graphs represented by Kappa strings. These are particular patterns (before and after the reaction event) that could be present in a simulation's *mixture* of explicitly represented protein agents (which form the set of complexes at any given time)(3; 7). At each event, a particular rule is selected with a probability proportional to the number of LHS pattern matches in a mixture and the rule's rate constant. During simulation,

KaSim can output specified observables (typically the number of matches of a Kappa string) at uniform increments of time. In addition to this time-course data we also employed the snapshot mechanism. This outputs the entire mixture (all present species) at a specified point in time. Both snapshots and observables were used in our analyses of the models.

For more information on the Kappa language itself, see (3; 7); descriptions of the simulation algorithm implemented in KaSim are present in (11; 20). Finally, KaSim itself is open-source software and can be downloaded at http://github.com/jkrivine/KaSim.

2.2 BioNetGen and NFsim

In order to confirm that our results were consistent with other rule-based modeling methods, we implemented the machine and ensemble model in the BioNetGen language (22). We see identical mean trajectories for NFsim and KaSim in Figs. S1 and S2 (additional comparisons can be seen in Section 3.1 and Figs. S4 and S5). We also employed the network generation tool present in BioNetGen to enumerate all the possible species that the machine model can create; this process failed for the ensemble model due to memory restrictions (see Section 3.5).

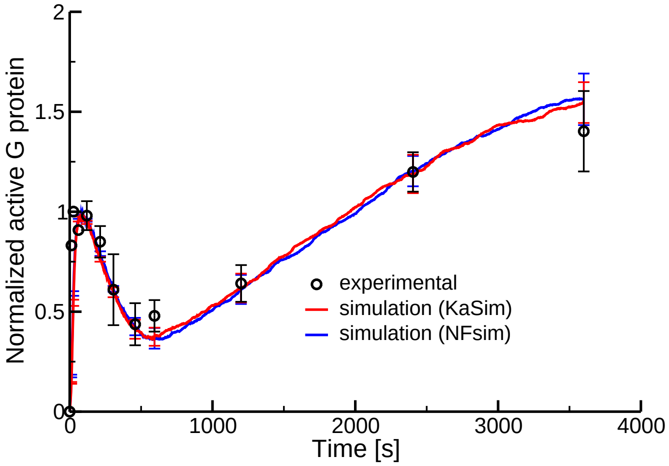

Figure S1: Comparison of G protein activation dynamics using NFsim and KaSim

2.3 Simulation methods

We employ a specific method for the simulation of our model in order to produce the most realistic results possible. Our method is outlined in Fig. S3. Since actual cells do not contain sets of monomers, we perform simulations in the absence of pheromone to generate an unstimulated *steady-state*. Specifically, we simulate our model for 1000 seconds starting from initial conditions which involve all agents in their monomeric state, aside from Gpa1 and Ste4, which are found in

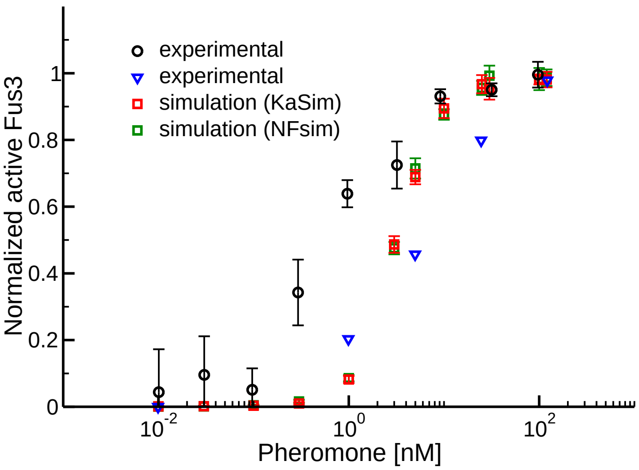

Figure S2: Comparison of dose-response trends using NFsim and KaSim

Gpa1/Ste4 heterodimers. Upon completion of N simulations, we output the mixtures as snapshots and use these sets of complexes as new sets of initial conditions. This simulated set of steady-states can be considered as representative of a population of N untreated yeast cells. For each steady-state, we add pheromone to induce the mating response (100 nM in all cases except the dose-response simulations) and then generate N' hour-long (in simulated time) trajectories, resulting in $N \times N'$ total signaling simulations.

For the drift calculations (3.3) we output snapshots on a logarithmic time-scale, and execute our pairwise comparisons between all simulations that originated from the same steady-state simulation (e.g. Fig. 3a in the main text has $N = 1$ and $N' = 10$ resulting in $\binom{N'}{2} = 45$ unique pairwise comparisons for each time point). This allows us to observe the heterogeneity among complexes generated solely from signaling (addition of pheromone), rather than from differences present before pheromone stimulation.

2.4 Parameter randomization

To examine the robustness of drift with respect to the chosen parameters, we generated 1000 different parameter sets for the ensemble and machine models in a manner similar to prior studies (23). For all non-varied and inferred or estimated rate parameters, the particular value was multiplied by a uniformly sampled number, x: $10^{-1} < x < 10^{1}$. For those parameters varied (seen above in red, 1.2) as well as those directly observed, the rate was also multiplied by a uniformly sampled number, but on a smaller range: $2^{-1} < x < 2^{1}$. This was done to maintain a level of realism in these randomizations, as the varied parameters typically have more influence over the experimentally determined time-course trends. Despite this, there was still noticeable deviation from wild-type behavior in these simulations (both time-course and dose-response) due to the

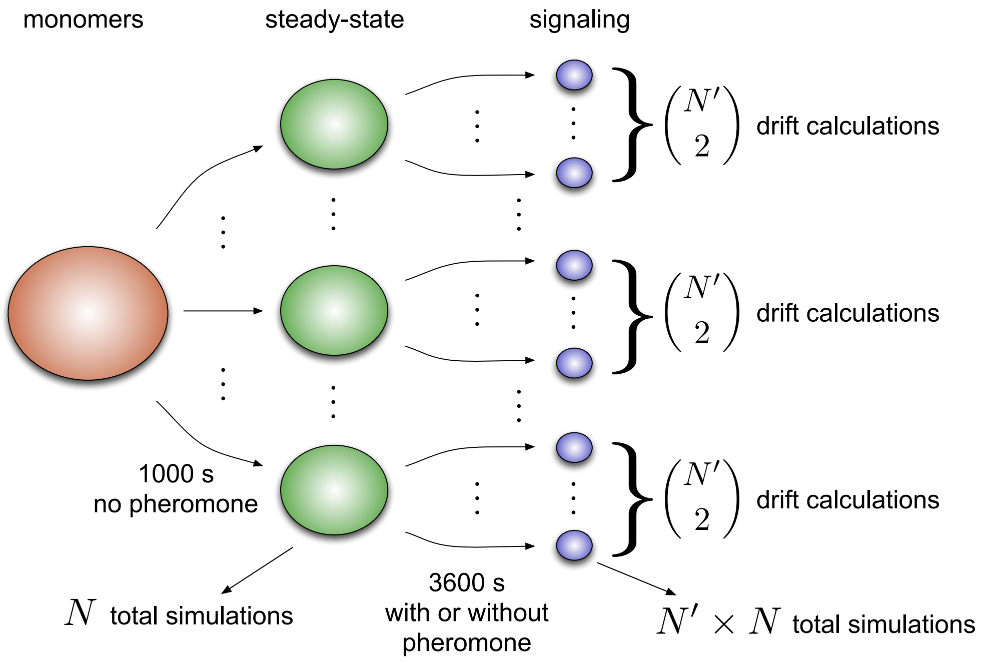

Figure S3: General method for calculating drift. A rule set is initialized with a specific set of proteins (red) and simulated for 1000 seconds to N independent steady-states (green). The steady-states are then used to generate N' signaling simulations each (blue, 1 hour of simulated time each), resulting in a total of $N \times N'$ simulations from the steady-state conditions for a particular rule or parameter set. Pairwise drift calculations are only performed between simulations with identical initial conditions, thus for each steady-state we have $\binom{N'}{2}$ drift values resulting in a total of $N \times \binom{N'}{2}$ drift points for any specific rule set. Note that we also performed the "signaling" simulations *without* pheromone to observe the baseline levels of drift in our model (seen in the main text, Fig. 3a).

wide range of parameter variation.

Upon generation of these parameter sets, each was simulated to $N = 3$ unique steady-states (Fig. S3). Subsequently, pheromone was added and $N' = 3$ trajectories were simulated, resulting in nine simulations of the signaling network for each unique parameter set. Thus for each set, we have three sets of three drift values, resulting in 9000 total drift points for the ensemble model, and slightly fewer (7789) for the machine model as simulation pairs where $d(i, j) = 0$ were ignored; these cases represented parameter sets that had essentially no signaling activity. Figs. 3b and 5b in the main text show this density distribution alongside distributions of drift values from the validated ensemble and machine parameter sets. In order to generate these distributions we used 50 copies of the final (ensemble or machine) model instead of 1000 unique parameter sets, resulting in 450 drift points (again, $N = 3$ and $N' = 3$). These densities were generated using

kernel density estimation (KDE) in the R statistical suite (24). We determined significant differences between the means of such distributions via a non-parametric two-tailed permutation test with 10^5 replications (also in R (24)).

3 Additional results

3.1 Model validation

Since each simulation requires approximately 3-4 hours of CPU time, standard methods of fitting our model to data (e.g. regression techniques) could not be implemented(25; 26). We thus manually varied parameters in the model in order to match experimental data. To do this, we identified a subset of parameters that govern experimentally observed trends(10; 27; 28; 29). We modified these parameters iteratively, within a biologically realistic range (1.2), to achieve reasonable overlap with experimental observation. It is important to note that the model reproduces these observations employing mass-action kinetics and does not utilize simplified Michaelis-Menten functions(2). Though the model reproduces the time- and pheromone-dependent trends of the yeast pheromone response cascade, it is clearly not the only possible solution, since we were able to construct a machine-like model which also replicates certain experimental trends (3.2).

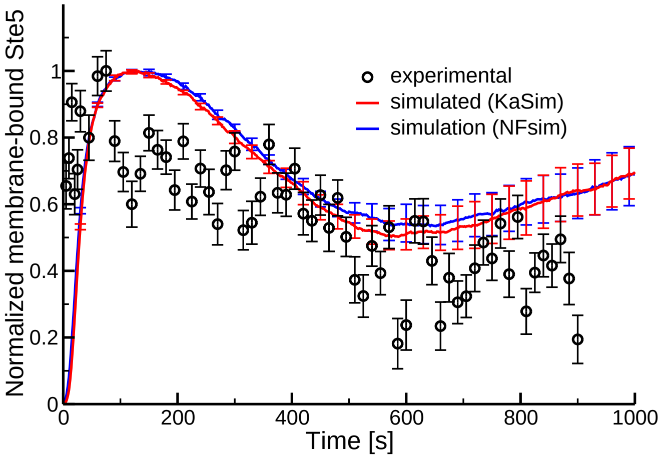

Figure S4: Activation of the pheromone cascade in the ensemble model results in rapid localization of the scaffold, Ste5, to the membrane as indicated by FRET measurements(27). Values are seen for the first 1000 seconds and the error bars represent 95% confidence intervals for both experimental and simulated data ($n = 3$ and $n = 10$, respectively).

The two graphs seen here (Fig. S4 and Fig. S5), in addition to those in the main text (Fig. 2), are the experimental trends(27; 28) that were used to validate our model. Our data is broadly consistent with experimental data (e.g. the initial spike in Ste5 membrane-recruitment in Fig. S4),

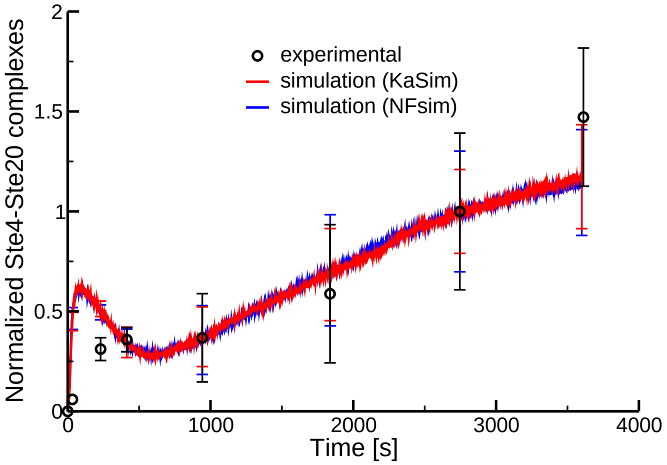

Figure S5: Fold increase over the basal number of Ste4-Ste20 dimers in the ensemble model (28). The error bars represent 95% confidence intervals for both experimental and simulated data ($n = 3$ and $n = 10$, respectively).

especially when considering the noise present in the experimental measurements and the potential impacts of photobleaching (27). Note that of the four sets of experimental data (including those described in the main text), two are directly affected by the concentration of unbound Ste4 (i.e. not bound to Gpa1): Ste4-Ste20 binding and active G-protein. Thus among the most influential unknown and varied parameters were the Ste12-induced synthesis rates of Ste4 and Gpa1.

3.2 Machine model validation

Validation of the machine model was accomplished in essentially the same way as validation of the ensemble model. Only very minor adjustments to the rates were needed to reproduce the G-protein temporal dynamics (Fig. S6), and the dose-response curve was readily matched upon altering the cooperativity of the Ste5 nuclear export rate (1.8, Fig. S7). Though not as accurate as the ensemble model, a similar trend for Ste4-Ste20 binding was seen in the machine model (Fig. S8). However, it was unable to exactly reproduce the behavior seen in Fig. S4. This was most likely due to the altered rules, which require Ste5 and its binding partners to remain membrane-bound in order to phosphorylate Fus3.

3.3 Compositional drift

Compositional drift was first introduced as a measure of intracellular heterogeneity in (11). Drift (d) is a pairwise comparison between the set of complexes, C, of two independent simulations, i and j, which originated from the same initial conditions. It is defined as the symmetric difference

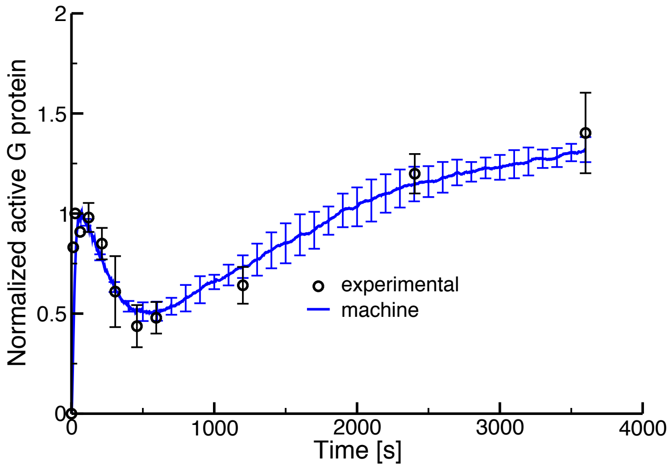

Figure S6: G-protein activation dynamics in the machine model (10). Error bars are 95% confidence intervals, experimental data is seen in black ($n = 3$), and simulations are seen in blue ($n = 10$).

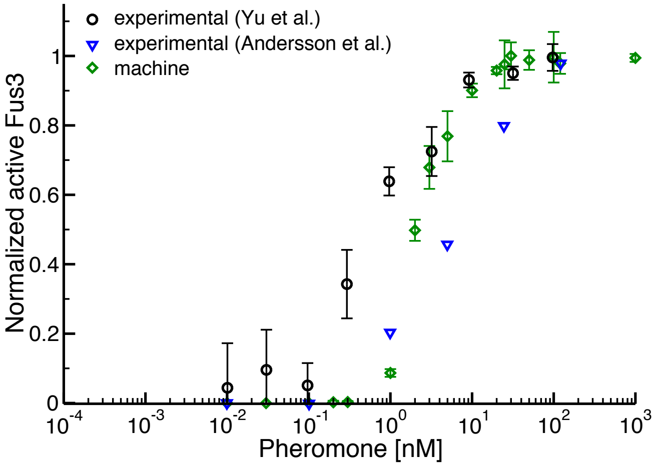

Figure S7: Dose-response dynamics in the machine model (phosphorylated Fus3 with respect to pheromone)(27; 29). Error bars are 95% confidence intervals. Data from (27) ($n = 3$) and (29) ($n =$ unknown) are in black and blue, respectively. Simulated data is in green ($n = 10$).

of the two sets divided by the union of the two sets:

$$d(i, j) = \frac{|C_i \,\Delta\, C_j|}{|C_i \cup C_j|}$$

where $|X|$ is the number of elements in some set X. This results in a normalized value between 0 and 1 where $d = 0$ indicates identical sets of complexes and $d = 1$ indicates disjoint sets. Given two simulated cells and their constituent complexes, drift is thus the probability that a given complex is present in one cell but not the other.

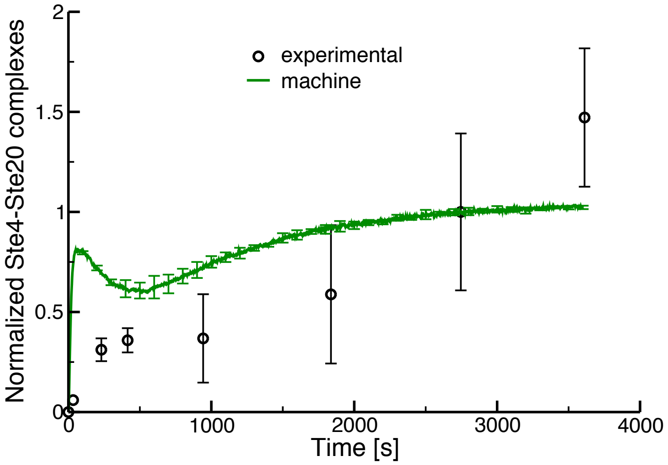

Figure S8: Fold increase over the basal number of Ste4-Ste20 dimers in the machine model. (28) The error bars represent 95% confidence intervals for both experimental and simulated data ($n = 3$ and $n = 10$, respectively).

As mentioned in the main text, this calculation takes into account any difference between two complexes, however minor. To confirm that this is a reasonable method of determining the level of heterogeneity among signaling species, we examined a number of different criteria. First, we took snapshots from ten simulations and calculated drift while ignoring any difference due to post-translational modifications (e.g. phosphorylation). We can see that although drift is reduced slightly in this case, substantial heterogeneity still exists when solely considering the binding patterns of the present complexes (Fig. S9).

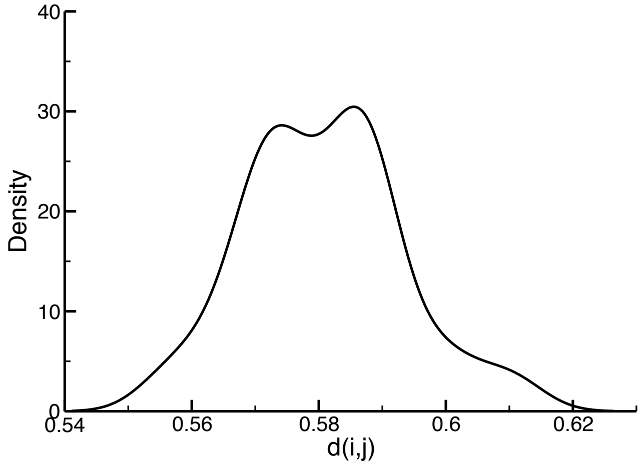

Figure S9: Drift density in the ensemble model at $t = 360$ seconds without consideration of post-translational modification ($n = 45$). The density was estimated using standard KDE methods in R (24)

However, phosphorylation is certainly important in a signaling cascade; Fus3 cannot induce

transcription of mating-related genes without being phosphorylated on two of its residues. Thus we investigated a comparison that can incorporate these distinctions while ignoring differences among complexes which have no behavioral consequence for the system. We used our ten snapshots to construct classes of complexes that are *functionally equivalent*. For two complexes to be functionally equivalent, the system (or rule set in our case) must not be able to distinguish between the complexes. In essence, one could exchange these two complexes without having any effect on the behavior at that point in time. We defined these equivalence classes using our rule set. In order to determine which reaction should take place next, each rule is assigned a stochastic rate of being chosen, called the *activity*(20). The activity is the product of the rate constant and the number of complexes in the mixture that match the LHS of the rule. The number of matches is important for a specific reason: if we have a rule where A converts to B at a rate k and there is no A in the mixture, the system cannot execute this rule regardless of its rate, thus the rule's activity is 0. By calculating the activity of every rule with respect to a particular complex, we obtain a signature for this complex within a particular rule set. If two (or more) complexes exhibit the same signature (i.e. every rule has the same activity) then they belong to the same equivalence class, because they exert the same influence on the system. We found that no two complexes were functionally equivalent over a set of 10 simulations, indicating that the structural distinctions included in our original definition of drift are functionally relevant (11).

In addition to examining the level of drift during peak signaling (Figs. 3b and 5b in the main text) we also examined drift over the union of logarithmically distributed time points. To do this, we compiled a list of the observed unique species from all time points for a particular simulation and performed the pairwise drift calculation with a similarly compiled list of unique species from another simulation. We found very similar results when calculating the density for $N = 50$ simulations (450 total drift values; Fig. S10), as compared to the drift densities at the single peak signaling time point ($t = 360$). This confirms that the large differences in generated species between simulations is not an artifact of choosing a single time point for the calculation.

We also investigated the rate at which a particular simulation diverged from its initial conditions based on drift. This was termed "autodrift," and is defined as the drift between a simulated cell i's sets of complexes at two different points in time: $d_i(t, t + \Delta t)$. We fit the data to an exponential function using standard nonlinear least-squares regression in R (24). Analysis of the residuals indicated that a single exponential fit did not capture the trend in the data. We therefore attempted fits using both double and triple exponential functions. The functional form of the full model is:

$$d_i(t, t + \Delta t) = \beta_1 - \beta_2 \cdot e^{-\beta_3 \Delta t} - \beta_4 \cdot e^{-\beta_5 \Delta t} - \beta_6 \cdot e^{-\beta_7 \Delta t}.$$

We found that fitting the entire model yielded an estimate for the third exponential term (i.e.$\hat{\beta}_7$) that was not statistically significant when correcting for multiple hypothesis testing. All the other estimates in all of the variants of the model were significant (see table below). Among this set of nested models we thus selected the double exponential fit as the one that most significantly describes the data. Close inspection of the residuals indicates that there may indeed be a significant further increase in drift on longer time scales (Fig.3c, main text); the difficulty in this case is that the time window is not long enough to capture this trend in a significant way. Longer simulations could thus yield a model where $\hat{\beta}_7$ is more significant. It is likely that these longer

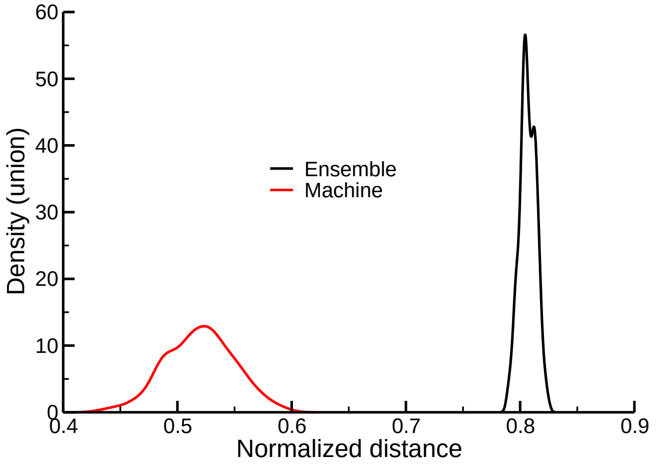

Figure S10: Drift density for scaffold-based signaling species over multiple, logarithmically distributed time points. Similar to Figs. 3B and 5B in the main text, we see a stark difference between the machine and ensemble models' average drift. Clearly the drift between simulations is not a result of the time point for which the drift calculation is made.

timescale changes in the value of drift represent more than just the turnover of transient complexes, but rather susbstantive changes in the system that arise due to the progress of the signal down the cascade.

**Parameters**	**Model Estimates (p-value)**		
	**single**	**double**	**triple**
$\hat{\beta}_1$	0.7633 ($< 2 \times 10^{-16}$)	0.7759 ($< 2 \times 10^{-16}$)	0.7850 ($< 2 \times 10^{-16}$)
$\hat{\beta}_2$	0.6821 ($< 2 \times 10^{-16}$)	0.2378 ($< 2 \times 10^{-16}$)	0.2693 ($< 2 \times 10^{-16}$)
$\hat{\beta}_3$	5.452 ($< 2 \times 10^{-16}$)	2.090 ($< 2 \times 10^{-16}$)	2.609 ($< 2 \times 10^{-16}$)
$\hat{\beta}_4$	N/A	0.5252 ($< 2 \times 10^{-16}$)	0.4849 ($< 2 \times 10^{-16}$)
$\hat{\beta}_5$	N/A	10.82 ($< 2 \times 10^{-16}$)	11.65 ($< 2 \times 10^{-16}$)
$\hat{\beta}_6$	N/A	N/A	0.02013 (4.98×10^{-16})
$\hat{\beta}_7$	N/A	N/A	0.01795 (0.005)

3.4 Species classification and clustering

Since most of the combinatorial complexity in this cascade is centered around Ste5 and all its potential interaction partners, we classified the complexes present in our snapshots into 6 categories or *bins*. In order to do this we constructed the largest possible complexes starting from monomers based on the rule set. The resulting 6 complexes (with Fus3 and Kss1 considered interchangeable) are the basis for classification; any species generated during simulation will

match a pattern present in one of these complexes and thus belong to its bin.

Three of the six bins are directly related to specific aspects of the response network: one bin contains all G-protein related complexes (and the pheromone peptide), another contains all species localized in the nucleus, and a third contains all scaffold-based species (note that some monomeric species could be placed in multiple bins: we placed these in the scaffold-based bin by convention). The remaining three bins are primarily composed of a kinase (Ste11, Ste7 and Fus3 and Kss1) and its specific phosphatase. Here we focus almost exclusively on the scaffold bin. A representation of the complex defining this bin is seen in Fig. S11.

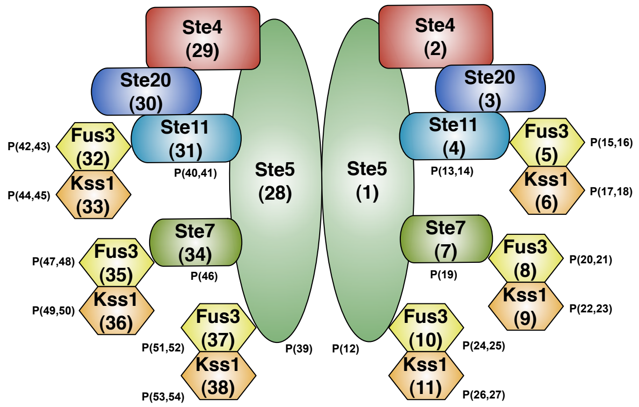

Figure S11: This diagram shows a labeling system for creating the integer sequences for the clustering of complexes (take note that this complex can never actually exist in our simulations since Fus3 and Kss1 cannot simultaneously bind their shared substrates; it is merely a visual representation of how we create unique identifiers for each complex). Each position in the sequence is associated either with a protein agent or an agent's phosphorylation site. Note that some agents have multiple phosphorylation sites (e.g. in our model, Fus3 can be phosphorylated on two independent residues). Each number in the sequence can have a range of integer values. For protein agents this is either 0 or 1, indicating their presence in the complex. The range of potential integers for phosphorylation sites vary between sites, however 0 indicates no phosphorylation for all sites. Sites representing a specific residue are either 1 or 0 (indicating the presence/absence of phosphorylation), however sites representing multiple residues can have values larger than this (e.g. Ste11 has a site representing 3 distinct residues requiring phosphorylation for its activation, therefore this site can contain values up to 3).

Following our analysis of subgraph conservation among scaffold-based signaling species, we performed clustering analysis on this subset of complexes during peak signaling ($t = 360$ seconds). This enabled us to formalize our search for the existence of a core complex. In order to cluster the complexes generated by our simulations we converted each complex in a particular snapshot to a unique sequence of integers (vector) in order to calculate the *graph edit distance* (G_{edit}) between any two species in the same bin. In graph theory, G_{edit} is the minimum number of *edits* or changes necessary to convert one graph to another. In the case of our depictions of macromolecular complexes, bonds are equivalent to edges (simple contact, with Ste20/Ste11 contact an exception) and protein or gene agents represent nodes. When considering these complexes in our vector notation, G_{edit} is the sum of the absolute value of differences at each

position in two sequences, r and s, of length, l:

$$G_{edit} = \sum_{n=0}^{l} |r_n - s_n|.$$

This is also known as the "Manhattan distance", or L^1-norm. We included differences in phosphorylation states in G_{edit}, as these differences clearly have an effect on the signaling network (3.3). In Fig. S11, we outline our method for creating these sequences. Note the symmetry in this scaffold-based bin, resulting in two possible integer sequences per complex and thus two unique ways to calculate G_{edit} between two particular species (as mentioned above, G_{edit} is always the minimum value in this situation). A sample calculation is seen in Fig. S12.

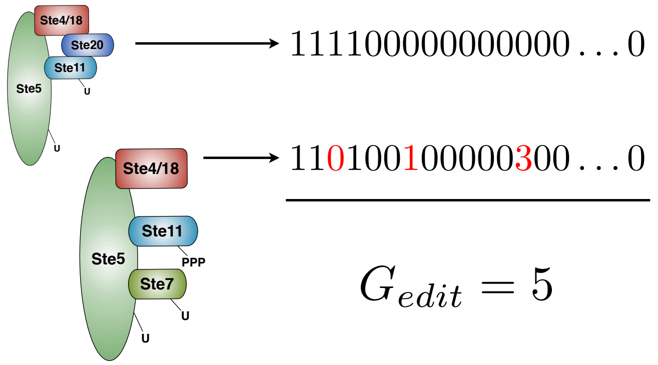

Figure S12: Calculating G_{edit} between two complexes. Each sequence is one of two potential vector representations for its complex. In terms of *graph edits*, we can see that there are five (seen in red): removal of Ste20, addition of unphosphorylated Ste7, and addition of three phosphates on Ste11.

Upon generation of these sequences, we proceeded to hierarchically cluster the complexes according to the G_{edit} matrix. We focused on clustroid-based single-linkage clustering for our data though both standard single- and complete-linkage criteria gave similar results. We also attempted to find the optimal number of clusters, N, via a specific stopping criterion, $E(i)$ (30). Unfortunately this criterion did not return consistent results between multiple snapshots, thus we chose $N = 10$ as our ultimate number of clusters for analysis. Varying this cutoff did not influence the results discussed below (seen below and in Fig. 4b in the main text).

If the complexes expressed some sort of conserved binding pattern, we would expect that the clusters generated from one snapshot would be near identical or similar to the clusters generated from another. However this was not the case, as the intercellular minimum between-cluster distance (MBCD) is nearly as large as the intracellular MBCD (when hierarchically clustering the complexes, the intercellular MBCD is the criteria for selecting which two clusters will join next, Fig. S13).

We also found that clusters containing more than 10 complexes exhibited very little conservation in terms of structural similarity between their constituent species. Note that in many of these clusters not even Ste5 dimers were conserved. In fact, the mean of the average G_{edit} between the clustroid and its cluster constituents (where the number of constituents ≥ 10) is greater than 6 as

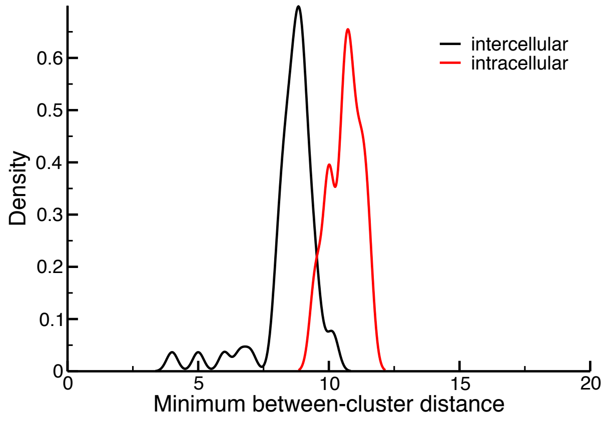

Figure S13: MBCD distributions for clusters in the ensemble model both between and within snapshots ($n_{intra} = 90$ and $n_{inter} = 45$). Though the mean intercellular MBCD is lower than the mean intracellular MBCD ($p < 10^{-5}$) we would expect that the intercellular MBCD would be near 0 if there was conservation within the scaffold species. Both densities were estimated using KDE methods in R (24)

seen in Fig. S14. A different way of framing this result is to consider the largest conserved component within a particular cluster (while still retaining the condition on the number of constituents). We calculate this conserved component using exclusively those entries in the vector representation that refer to protein presence/absence; we do not consider phosphorylation state. With our standard clustering cutoff of $N = 10$ (Fig. S15, black) we see a distribution where a conserved component consisting of only 2 proteins is in the 89th percentile. As we increase N, the mean of this distribution changes slightly, but maintains consistently low values (near or around 2). We also see a shift in the peak of the distribution (from 0 to 2) between $N = 10$ and $N = 20$, however it remains at 2 through $N = 100$ indicating consistently low conservation within clusters of reasonable size. Thus we reach the same conclusion with our clustering that we did with our simpler analysis of structural conservation seen in Fig. 4A in the main text.

3.5 Enumerating all possible species

In order to determine the total number of scaffold-based (i.e. Ste5-bound) protein complexes that can possibly form, we proceeded to use BioNetGen to construct the reaction network for the two models. The output listed all species that could be formed from that particular ruleset, and from this list we found a total of 1106 Ste5-bound species for the machine model. BioNetGen was incapable of enumerating the species present in the ensemble model due to memory restrictions, so we analytically calculated the total number of scaffold-based species. We implemented a counting procedure made relatively simple by the mutual independence of many of the binding interactions in the ensemble model (i.e. Ste5 can bind Ste11 independent of all of Ste5's other binding sites and independent of Ste11's phosphorylation state). Consider the structure seen in Fig. S11. Since all of Ste5's binding interactions are mutually independent we can focus on each of Ste5's sites individually and then take the product across all sites to estimate the total number of possible states. Initially we will focus on molecules that have *only one* Ste5.

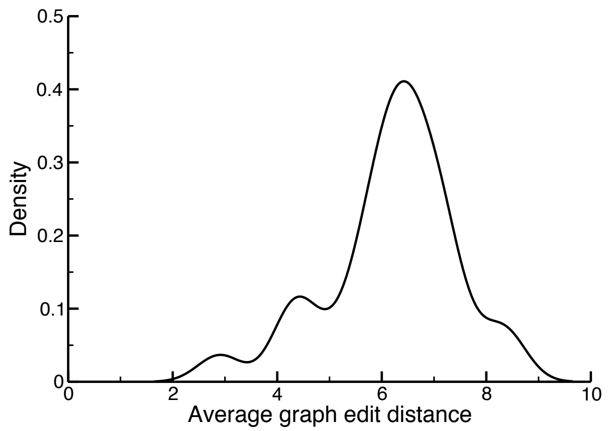

Figure S14: Distribution of average G_{edit} scores between a clustroid and its constituent complexes in the ensemble model ($N = 26$). Note this is only calculated when the number of elements in a cluster is ≥ 10. Upon consideration of 10 independent simulations (and their snapshots) only 26 of the total 100 clusters contained over 10 complexes. Density estimated using KDE methods in R (24)

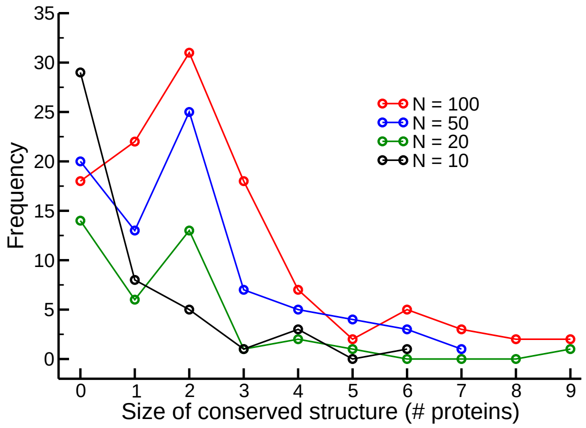

Figure S15: Frequency of the size of the largest conserved component in clusters with 10 or more complexes. Different colors represent different distributions based on varying values of the clustering cutoff, N. Total number of clusters for each distribution: red = 110, blue = 78, green = 38, black = 47.

We see that Ste5's top site binds Ste4 and Ste4 can bind Ste20. This results in 3 total species, assuming Ste5 is always present: Ste5, Ste5-Ste4, and Ste5-Ste4-Ste20, since Ste20 can only be present in the presence of Ste4. The second site on Ste5 binds Ste11 (again, independently of all other sites), and Ste11 can be in 4 phosphorylation states according to the agent declaration present in the Kappa model file:

%agent: Ste11(mapk, ste5, degradation~u~p, S302_S306_S307~u~p~pp~ppp)

Since we are primarily concerned with those structures directly related to signal transduction, we will ignore the 'degradation' site for this calculation. Thus we have 5 different states (Ste5 unbound + 4 Ste5-Ste11 states). Fig. S11 also shows the possibility of Fus3 *or* Kss1 binding to Ste11, and this interaction is independent both from Ste11's phosphorylation state and Fus3/Kss1's phosphorylation state. Since both Fus3 and Kss1 can be in 4 phosphorylation states, we have a total of 9 possible ways that Ste11 can bind a MAPK while bound to Ste5 (Ste5-Ste11 unbound + 4 Ste5-Ste11-Fus3 states + 4 Ste5-Ste11-Kss1 states). Finally, we multiply the 4 Ste5-Ste11 states times the 9 Ste5-Ste11-MAPK states and add the remaining unbound Ste5, resulting in 37 total states for Ste5's Ste11 binding site.

Further counting is as follows:

**Ste5 site**	**States**	**Description**
Ste4	3	Ste5 + Ste5-Ste4 + Ste5-Ste4-Ste20
Ste11	37	4 Ste11 states · 9 MAPK states + 1 unbound Ste5
Ste7	28	3 Ste7 states · 9 MAPK states + 1 unbound Ste5
MAPK	9	8 MAPK states + 1 unbound Ste5
phosphorylation	2	2 phosphorylation states
Total	55944	

From here we can then enumerate all species that include a Ste5 molecule. Since both Ste5 molecules can operate independently we have 55944^2 species with Ste5 dimers + 55944 species with a Ste5 monomer, resulting in nearly 3.13 billion Ste5-based species, nearly a 3 million-fold increase compared to the machine model.

3.6 Socio-affinity scoring

We used the socio-affinity (SA) index described in (31) to determine whether standard methods of deriving complex information from high-throughput data, such as tandem affinity purification (TAP), can distinguish between machine-like complexes and ensembles of signaling species. Note that the following definitions and equations merely summarize descriptions given in (31). The SA score between protein i and j, $A(i,j)$, is based on binary (bait and prey) interaction data, and is a linear combination of a number of terms:

$$A(i,j) = S_{i,j|i=bait} + S_{i,j|j=bait} + M_{i,j}$$

where

$$S_{i,j|i=bait} = \log\left(\frac{n_{i,j|i=bait}}{f_i^{bait} \cdot n_{bait} \cdot f_j^{prey} \cdot n_{i=bait}^{prey}}\right), \quad M_{i,j} = \log\left(\frac{n_{i,j}^{prey}}{f_i^{prey} \cdot f_j^{prey} \cdot \sum_{all-baits} n_{prey} \cdot \frac{(n_{prey}-1)}{2}}\right).$$

For the terms in $S_{i,j|i=bait}$ we have the following: $n_{i,j|i=bait}$ is the number of j's that i pulls down when i is bait, f_i^{bait} is the frequency that i was bait, n_{bait} is the number of unique bait proteins, f_j^{prey} is the fraction of times that j was pulled down by any bait that was not j itself, and $n_{i=bait}^{prey}$

is the total number of proteins i pulled down not counting itself. Since we are performing TAP *in silico*, we are guaranteed to retrieve all prey proteins for a particular bait and do not need multiple replications with the same bait (as all preys are explicitly accounted for in the snapshot). This means that $f_i^{bait} = \frac{1}{n_{bait}}$ and the S term simplifies to:

$$S_{i,j|i=bait} = \log\left(\frac{n_{i,j|i=bait}}{f_j^{prey} \cdot n_{i=bait}^{prey}}\right).$$

This term is thus the logarithm of the number of times i pulls down j, divided by the expected value of this number (which is the total number of times j is pulled down times the total number of proteins i pulls down as bait).

The terms in $M_{i,j}$ are as follows: $n_{i,j}^{prey}$ is the number of "purifications" in which i and j are observed together when neither i nor j are bait, f_i^{prey} and f_j^{prey} are the fraction of unique monomers pulled down by i or j, respectively, and n_{prey} is the number of unique monomers pulled down with bait i. Note that this last term is summed over all bait proteins, and thus does not require an index. $M_{i,j}$ is thus the logarithm of the observed "co-purifications" of i and j over its expected value, which is the frequency of observing i and j together over all baits when neither i nor j are baits.

We created an $N \times N$ matrix of SA scores over all protein types ($N = 18$ unique proteins) for snapshots generated during peak signaling ($t = 360$ s) in both the machine and ensemble models. As this is a symmetric matrix, there are 153 unique pairings of proteins (and thus 153 SA scores). The majority of these pairings result in a score of 0 since there is no possibility of their presence in the same complex (e.g. Pheromone and Dig1). It is plain to see, however, that the SA scores which do exist are between proteins that are in the same bins as discussed above in Section 3.4. The ensemble model's SA matrix is divided over two tables (Table 1 and Table 2); some interactions' SA scores are shown twice (e.g. Ste11 and Ste7) and the scores not shown are equal to 0. Fig. 6a in the main text shows the correlation between the values in the machine and ensemble SA matrices.

We can then create clusters or "functional modules" as referred to in (32) using the Markov clustering (MCL) algorithm outlined in (33). The MCL algorithm partitions the set of proteins into disjoint clusters based on their SA scores, yet we know that certain proteins may associate with multiple types of complexes (e.g. Ste4 associates with G-protein related proteins and scaffold related proteins). To allow these proteins to be "shared" between modules we adapted Pu *et al.*'s method (32) and checked for proteins that had interactions with proteins in a distinct cluster. If a protein has positive SA scores with 75% of those in the "acceptor" cluster, we consider it a member of the acceptor cluster in addition to its original cluster. Representative clusters can be seen in Fig. 6a in the main text.

	**Phe.**	**Ste2**	**Sst2**	**Gpa1**	**Ste4**	**Ste20**	**Ste5**	**Ste7**	**Ste11**	**Ste12**	**Dig1**	**Dig2**	**Fus3**	**Kss1**
**Phe.**	0	5.1048	4.6352	4.2624	2.7848	0	0	0	0	0	0	0	1.8951	1.1902
**Ste2**	5.1048	0	4.6607	4.2888	2.8293	0	0	0	0	0	0	0	1.918	1.2127
**Sst2**	4.6352	4.6607	0	3.818	2.4891	0	0	0	0	0	0	0	2.9292	2.213
**Gpa1**	4.2624	4.2888	3.818	0	4.7597	0	0	0	0	0	0	0	1.0759	0.3699
**Ste4**	2.7848	2.8293	2.4891	4.7597	0	3.7073	3.5454	1.6443	2.8402	0	0	0	1.8825	1.3433
**Ste20**	0	0	0	0	3.7073	0	7.1188	5.1865	6.3936	0	0	0	3.6351	3.1986
**Ste5**	0	0	0	0	3.5454	7.1188	0	5.9115	7.0845	0	0	0	4.2624	3.8706
**Ste7**	0	0	0	0	1.6443	5.1865	5.9115	0	5.1393	0	0	0	4.4198	5.2097
**Ste11**	0	0	0	0	2.8402	6.3936	7.0845	5.1393	0	0	0	0	4.0232	3.6254
**Ste12**	0	0	0	0	0	0	0	0	0	0	9.2719	8.7543	3.939	5.0572
**Dig1**	0	0	0	0	0	0	0	0	0	9.2719	0	8.9105	3.8773	4.7526
**Dig2**	0	0	0	0	0	0	0	0	0	8.7543	8.9105	0	3.805	4.2255
**Fus3**	1.8951	1.918	2.9292	1.0759	1.8825	3.6351	4.2624	4.4198	4.0232	3.939	3.8773	3.805	0	1.5936
**Kss1**	1.1902	1.2127	2.213	0.3699	1.3433	3.1986	3.8706	5.2097	3.6254	5.0572	4.7526	4.2255	1.5936	0

Table 1: Socio-affinity score table for proteins associated with the G-protein cycle, scaffold-based signaling, and transcriptional regulation. The three blocks of nonzero values correspond to the bins described in Section 3.4 (note that the MAPKs are present all these bins).

	Ste7	Ste11	Mekp	Mekkp	Ptp	Msg5	Fus3	Kss1
**Ste7**	0	5.1393	9.711	0	0	0	4.4198	5.2097
**Ste11**	5.1393	0	0	9.7515	0	0	4.0232	3.6254
**Mekp**	9.711	0	0	0	0	0	3.5648	4.8434
**Mekkp**	0	9.7515	0	0	0	0	0.5554	0.1187
**Ptp**	0	0	0	0	0	0	2.5208	6.4925
**Msg5**	0	0	0	0	0	0	3.0833	6.4005
**Fus3**	4.4198	4.0232	3.5648	0.5554	2.5208	3.0833	0	1.5936
**Kss1**	5.2097	3.6254	4.8434	0.1187	6.4925	6.4005	1.5936	0

Table 2: SA score table for proteins associated with kinase regulation.

3.7 Robustness of combinatorial inhibition

In order to confirm that our results on combinatorial inhibition were not artifacts of the parameter sets of the machine and ensemble models, we created 100 models with randomized parameters for both the machine and ensemble model. The procedure for generation and simulation of these models follows that described in 2.4. We simulated these models at wild-type, 12x, and 60x concentrations of Ste5. We found that for all ensemble-based models, 12x concentrations of Ste5 increased Fus3 activation and 60x concentrations of Ste5 decreased Fus3 activation relative to the 12x activation level, confirming the robust presence of combinatorial inhibition (Fig. S16). The machine-based models display resistance to combinatorial inhibition (Fig. S17), with most models producing similar levels of Fus3 activation at 12x and 60x concentrations of Ste5.

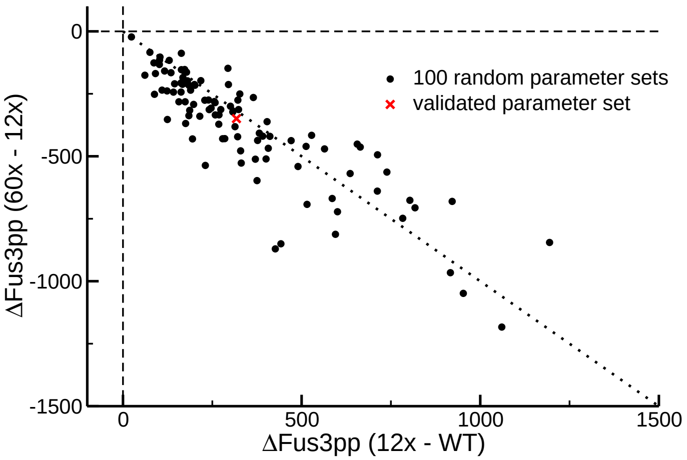

Figure S16: Ensemble parameter randomizations (100 parameter sets). The x-axis is the difference in Fus3 activation (in number of molecules) between WT and 12x concentrations of scaffold and y-axis is the difference in Fus3 activation between 12x and 60x concentrations. Dashed lines are $x = 0$ and $y = 0$ for reference, and the dotted line is $y = -x$ to accentuate the strong correlation between these values.

Calculation of relative ΔFus3pp values in Fig. 6c of the main text was performed by subtracting the number of active Fus3 molecules at peak signaling (Fus3pp) for some scaffold concentration from Fus3pp for some higher scaffold concentration. In the case of Fig. 6c in the main text, these values are 60x WT and 12x WT. This value was then divided by Fus3pp for the lesser of the two scaffold values. The resulting value is then a measure of the relative increase (> 1) or decrease (< 1) of the change in Fus3 activation during peak signaling.

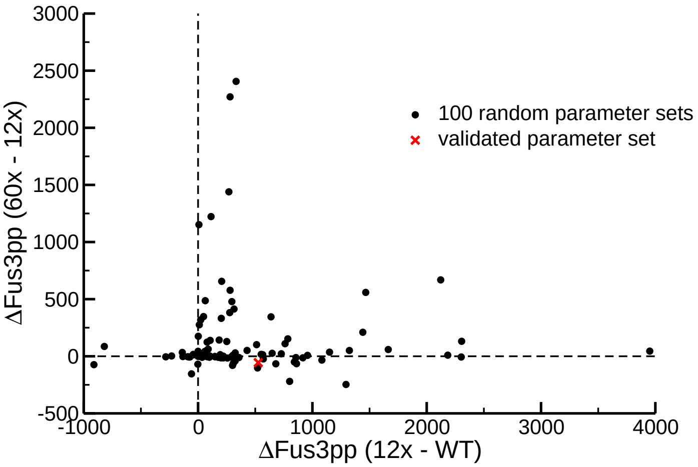

Figure S17: Machine prozone parameter randomizations (100 parameter sets). Axes and dashed lines are as Fig. S16

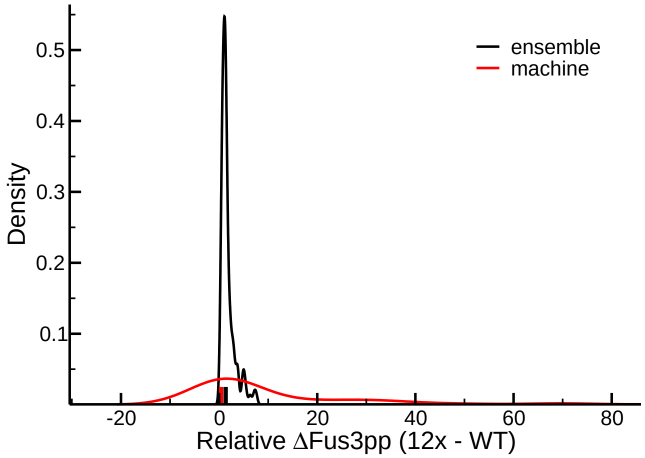

Figure S18: Relative ΔFus3pp values for randomized machine and ensemble models have similar means when considering the difference in scaffold concentration between 12x and wild-type values, but the machine model's distribution has a much higher variance. The majority of values in both models, however, were positive, indicating a general increase in signaling activity.

References

1. Sneddon MW, Faeder JR, Emonet T (2010) Efficient modeling, simulation and coarse-graining of biological complexity with NFsim. Nat Methods 8: 177–183.

2. Shao D, Zheng W, Qiu W, Ouyang Q, Tang C (2006) Dynamic studies of scaffold-dependent mating pathway in yeast. Biophys J 91: 3986–4001.

3. Danos V, Laneve C (2004) Formal molecular biology. Theoretical Computer Science 325: 69–110.

4. Danos V, Feret J, Fontana W, Harmer R, Krivine J (2007) Rule-Based Modelling of Cellular Signalling, volume 4703 of *Lecture Notes in Computer Science*. Berlin, Heidelberg: Springer Berlin Heidelberg.

5. Danos V, Feret J, Fontana W, Harmer R, Krivine J (2008) Rule-Based Modelling, Symmetries, Refinements, volume 5054 of *Lecture Notes in Computer Science*. Berlin, Heidelberg: Springer Berlin Heidelberg.

6. Danos V, Feret J, Fontana W, Krivine J (2008) Abstract interpretation of cellular signalling networks. Lecture Notes in Computer Science 4905: 83–97.

7. Feret J, Danos V, Krivine J, Harmer R, Fontana W (2009) Internal coarse-graining of molecular systems. PNAS 106: 6453–6458.

8. Ghaemmaghami S, Huh W, Bower K (2003) Global analysis of protein expression in yeast. Nature .

9. Thomson TM, Benjamin KR, Bush A, Love T, Pincus D, et al. (2011) Scaffold number in yeast signaling system sets tradeoff between system output and dynamic range. PNAS 108: 20265–20270.

10. Yi TM, Kitano H, Simon MI (2003) A quantitative characterization of the yeast heterotrimeric G protein cycle. PNAS 100: 10764–10769.

11. Deeds EJ, Krivine J, Feret J, Danos V, Fontana W (2012) Combinatorial complexity and compositional drift in protein interaction networks. PloS One 7: e32032.

12. Powell CD (2003) Chitin scar breaks in aged Saccharomyces cerevisiae. Microbiology 149: 3129–3137.

13. Chen RE, Thorner J (2007) Function and regulation in MAPK signaling pathways: lessons learned from the yeast Saccharomyces cerevisiae. Biochim Biophys Acta 1773: 1311–1340.

14. Inouye C, Dhillon N, Durfee T, Zambryski PC, Thorner J (1997) Mutational analysis of STE5 in the yeast Saccharomyces cerevisiae: application of a differential interaction trap assay for examining protein-protein interactions. Genetics 147: 479–492.

15. Bhattacharyya RP, Reményi A, Good MC, Bashor CJ, Falick AM, et al. (2006) The Ste5 scaffold allosterically modulates signaling output of the yeast mating pathway. Science 311: 822–826.

16. Maleri S, Ge Q, Hackett EA, Wang Y, Dohlman HG, et al. (2004) Persistent activation by constitutive Ste7 promotes Kss1-mediated invasive growth but fails to support Fus3-dependent mating in yeast. Mol Cell Biol 24: 9221–9238.

17. Mahanty SK, Wang Y, Farley FW, Elion EA (1999) Nuclear shuttling of yeast scaffold Ste5 is required for its recruitment to the plasma membrane and activation of the mating MAPK cascade. Cell 98: 501–512.

18. Roberts CJ, Nelson B, Marton MJ, Stoughton R, Meyer MR, et al. (2000) Signaling and circuitry of multiple MAPK pathways revealed by a matrix of global gene expression profiles. Science 287: 873–880.

19. Williamson JR (2008) Cooperativity in macromolecular assembly. Nat Chem Biol 4: 458–465.

20. Danos V, Feret J, Fontana W, Krivine J (2007) Scalable Simulation of Cellular Signaling Networks, volume 4807 of *Lecture Notes in Computer Science*. Berlin, Heidelberg: Springer Berlin Heidelberg.

21. Gillespie DT (1977) Exact stochastic simulation of coupled chemical reactions. J Phys Chem 81.

22. Hlavacek WS, Faeder JR, Blinov ML (2006) Rules for Modeling Signal-Transduction Systems – Hlavacek et al. 2006 (344): re6 – Science Signaling. Science (New York, NY) .

23. Faeder JR, Blinov ML, Goldstein B, Hlavacek WS (2005) Combinatorial complexity and dynamical restriction of network flows in signal transduction. Systems Biology 2: 5–15.

24. (2010) R: A Language and Environment for Statistical Computing. Vienna, Austria: The R Foundation for Statistical Computing.

25. Chen WW, Schoeberl B, Jasper PJ, Niepel M, Nielsen UB, et al. (2009) Input-output behavior of ErbB signaling pathways as revealed by a mass action model trained against dynamic data. Mol Syst Biol 5: 239.

26. Wang Y, Christley S, Mjolsness E, Xie X (2010) Parameter inference for discretely observed stochastic kinetic models using stochastic gradient descent. BMC Syst Biol 4: 99.

27. Yu RC, Pesce CG, Colman-Lerner A, Lok L, Pincus D, et al. (2008) Negative feedback that improves information transmission in yeast signalling. Nature 456: 755–761.

28. Leeuw T, Wu C, Schrag JD, Whiteway M, Thomas DY, et al. (1998) Interaction of a G-protein beta-subunit with a conserved sequence in Ste20/PAK family protein kinases. Nature 391: 191–195.

29. Andersson J, Simpson DM, Qi M, Wang Y, Elion EA (2004) Differential input by Ste5 scaffold and Msg5 phosphatase route a MAPK cascade to multiple outcomes. EMBO J 23: 2564–2576.

30. Xu S, Kamath MV, Capson DW (1993) Selection of partitions from a hierarchy. Pattern Recognition Letters 14: 7 - 15.

31. Gavin AC, Aloy P, Grandi P, Krause R, Boesche M, et al. (2006) Proteome survey reveals modularity of the yeast cell machinery. Nature 440: 631–636.

32. Pu S, Vlasblom J, Emili A, Greenblatt J, Wodak SJ (2007) Identifying functional modules in the physical interactome of Saccharomyces cerevisiae. Proteomics 7: 944–960.

33. van Dongen S (2000) A Cluster algorithm for graphs. Report - Information systems : 1–40.
